# Audiovisual Rehabilitation in Hemianopia: A Model-Based Theoretical Investigation

**DOI:** 10.3389/fncom.2017.00113

**Published:** 2017-12-15

**Authors:** Elisa Magosso, Cristiano Cuppini, Caterina Bertini

**Affiliations:** ^1^Department of Electrical, Electronic, and Information Engineering “Guglielmo Marconi”, University of Bologna, Cesena, Italy; ^2^Centre for Studies and Research in Cognitive Neuroscience, University of Bologna, Cesena, Italy; ^3^Department of Psychology, University of Bologna, Italy

**Keywords:** neurocomputational modeling, multisensory integration, Superior Colliculus, synaptic plasticity, visual rehabilitation, restitutive mechanisms, compensatory mechanisms

## Abstract

Hemianopic patients exhibit visual detection improvement in the blind field when audiovisual stimuli are given in spatiotemporally coincidence. Beyond this “online” multisensory improvement, there is evidence of long-lasting, “offline” effects induced by audiovisual training: patients show improved visual detection and orientation after they were trained to detect and saccade toward visual targets given in spatiotemporal proximity with auditory stimuli. These effects are ascribed to the Superior Colliculus (SC), which is spared in these patients and plays a pivotal role in audiovisual integration and oculomotor behavior. Recently, we developed a neural network model of audiovisual cortico-collicular loops, including interconnected areas representing the retina, striate and extrastriate visual cortices, auditory cortex, and SC. The network simulated unilateral V1 lesion with possible spared tissue and reproduced “online” effects. Here, we extend the previous network to shed light on circuits, plastic mechanisms, and synaptic reorganization that can mediate the training effects and functionally implement visual rehabilitation. The network is enriched by the oculomotor SC-brainstem route, and Hebbian mechanisms of synaptic plasticity, and is used to test different training paradigms (audiovisual/visual stimulation in eye-movements/fixed-eyes condition) on simulated patients. Results predict different training effects and associate them to synaptic changes in specific circuits. Thanks to the SC multisensory enhancement, the audiovisual training is able to effectively strengthen the retina-SC route, which in turn can foster reinforcement of the SC-brainstem route (this occurs only in eye-movements condition) and reinforcement of the SC-extrastriate route (this occurs in presence of survived V1 tissue, regardless of eye condition). The retina-SC-brainstem circuit may mediate compensatory effects: the model assumes that reinforcement of this circuit can translate visual stimuli into short-latency saccades, possibly moving the stimuli into visual detection regions. The retina-SC-extrastriate circuit is related to restitutive effects: visual stimuli can directly elicit visual detection with no need for eye movements. Model predictions and assumptions are critically discussed in view of existing behavioral and neurophysiological data, forecasting that other oculomotor compensatory mechanisms, beyond short-latency saccades, are likely involved, and stimulating future experimental and theoretical investigations.

## Introduction

The primary human visual pathway conveys the majority of retinal fibers to the lateral geniculate nucleus of the thalamus and then, via the optic radiations, to the primary visual cortex (V1) (the retino-geniculo-striate pathway). V1 is the main distributor of visual information to extrastriate visual areas, for further processing. A secondary visual pathway (the retino-collicular pathway) routes a minority of retinal fibers directly to the Superior Colliculus (a midbrain structure), which also has reciprocal connections with striate and extrastriate visual cortices (May, 2006).

Patients with lateralized damages to the primary visual cortex (V1) or to the neural pathway feeding V1 often develop homonymous hemianopia, a visual field defect with the loss of conscious vision in one hemifield. Hemianopic patients cannot perceive visual stimuli presented in the blind hemifield; moreover, they show the inability to spontaneously develop effective oculomotor strategies to compensate for the visual field loss (Hildebrandt et al., 1999; Zihl, 2000; Tant et al., 2002).

Despite the visual deficit, hemianopic patients can preserve the ability to integrate audiovisual stimuli in the affected field, with beneficial effects (Frassinetti et al., 2005; Leo et al., 2008). In particular, data by Frassinetti and colleagues (Frassinetti et al., 2005) show that patients performing a visual detection task, while maintaining central fixation, significantly improved conscious visual detections in the affected field, when the auditory stimuli were applied in spatial and temporal coincidence with the visual targets.

The Superior Colliculus is the most likely structure mediating this multisensory improvement, because of its anatomical connections and the properties of its neuronal responses. Indeed, SC neurons receive not only visual information but also signals from other different sensory modalities, such as audition (Meredith and Stein, 1986; Stein and Meredith, 1993; May, 2006). Visual and auditory information are integrated in multisensory SC neurons according to specific principles (Stein and Meredith, 1993): an audiovisual stimulation elicits a stronger neuronal activation than each single component, when the visual and auditory components are presented in spatial and temporal register (spatial and temporal principle). Moreover, a proportionally greater enhancement of multisensory neuronal activation is evoked when weakly effective unisensory stimuli are combined, compared to the combination of highly effective stimuli (inverse effectiveness principle). The SC integrative principles have strong implications in hemianopia, as the SC and the retino-collicular pathway are preserved in these patients. Visual retinal input to SC, although weak, can still be efficiently combined with an accessory auditory input thanks to the inverse effectiveness principle, provided the rule of spatial and temporal proximity is satisfied. Furthermore, SC multisensory enhancement can affect cortical visual processing thanks to the projections from the SC to the visual cortices.

In addition to the immediate, “online” multisensory improvement in visual detection, there is also evidence of prolonged, “offline” effects that can be induced by repeated exposure to audiovisual stimuli. Indeed, long-lasting improvements of visual performances in hemianopic patients, promoted by audiovisual training protocols stimulating the blind hemifield, have been reported (Bolognini et al., 2005; Passamonti et al., 2009; Dundon et al., 2015b; Tinelli et al., 2015; Grasso et al., 2016). During the training, a visual target was given in close spatial and temporal proximity with an auditory stimulus, at various positions in the visual field; patients were asked to detect the presence of the visual target, by directing the gaze toward it from a central fixation point. Results revealed a significant post-training improvement in detection of unimodal visual targets in the blind field when the patients were allowed to use eye movements, while a weak amelioration was found when they had to maintain central fixation (Bolognini et al., 2005; Tinelli et al., 2015). Such results suggest that the audiovisual training could promote an increased oculomotor response to visual stimuli in the affected hemifield.

Beyond the “online” effects of audiovisual stimulation, the Superior Colliculus is a possible candidate for mediating the training effects, too. Indeed, the SC projects to brainstem motor areas controlling eyes and head orientation, and is critically involved in the initiation and execution of reflexive (i.e., exogenously-driven) saccades (Sparks, 1986; Jay and Sparks, 1987a; May, 2006; Johnston and Everling, 2008). Importantly, more than 70% of SC neurons projecting to the brainstem and, therefore involved in saccade generation, respond to multisensory stimulations (Meredith and Stein, 1986). As such, audiovisual stimuli, enhancing multisensory SC activation, might plastically reinforce the gain of the transduction from the SC sensory response to the motor output; in other words, after training the oculomotor system could have acquired increased responsiveness to the visual input conveyed via the retino-collicular pathway. However, the plastic mechanisms and synaptic reorganization that can functionally instantiate these visuomotor capabilities remain undetermined. Moreover, it is unclear whether the training may even stimulate genuine visual restitution beyond oculomotor compensation, and how the compensatory and restitutive effects may complementary contribute to visual improvements.

Recently, we have developed a neural network model (Magosso et al., 2016) that formalized the main cortico-collicular loops involved in audiovisual integration, and implemented—via neural connections and input-output neural characteristics—the SC multisensory integrative principles. The network postulated neural correlates of visual consciousness and mimicked unilateral V1 lesion. Simulations, performed in fixed-eyes condition, reproduced the “online” effects of enhanced visual detection under audiovisual stimulation.

Here, we extend our previous neural network to explore the effects of training in simulated hemianopic patients, providing quantitative predictions that can contribute to a mechanistic understanding of visual performance improvement observed in real patients. To this aim, the network has been integrated by novel elements. First, we have included a module of saccade generation, embracing the colliculus sensory-motor transduction; in this way, we can account for the potentiality of short-latency saccades triggered in a bottom-up fashion. Second, Hebbian mechanisms of synaptic learning have been implemented and adopted during training simulations. Different training paradigms (audiovisual multisensory/visual unisensory stimulation in eye-movements/fixed-eyes condition) are tested, to examine their efficacy in promoting different forms of rehabilitation (compensatory/restitutive), and to assess the predicted results in light of *in vivo* data.

## Materials and methods

**Figure 1:**
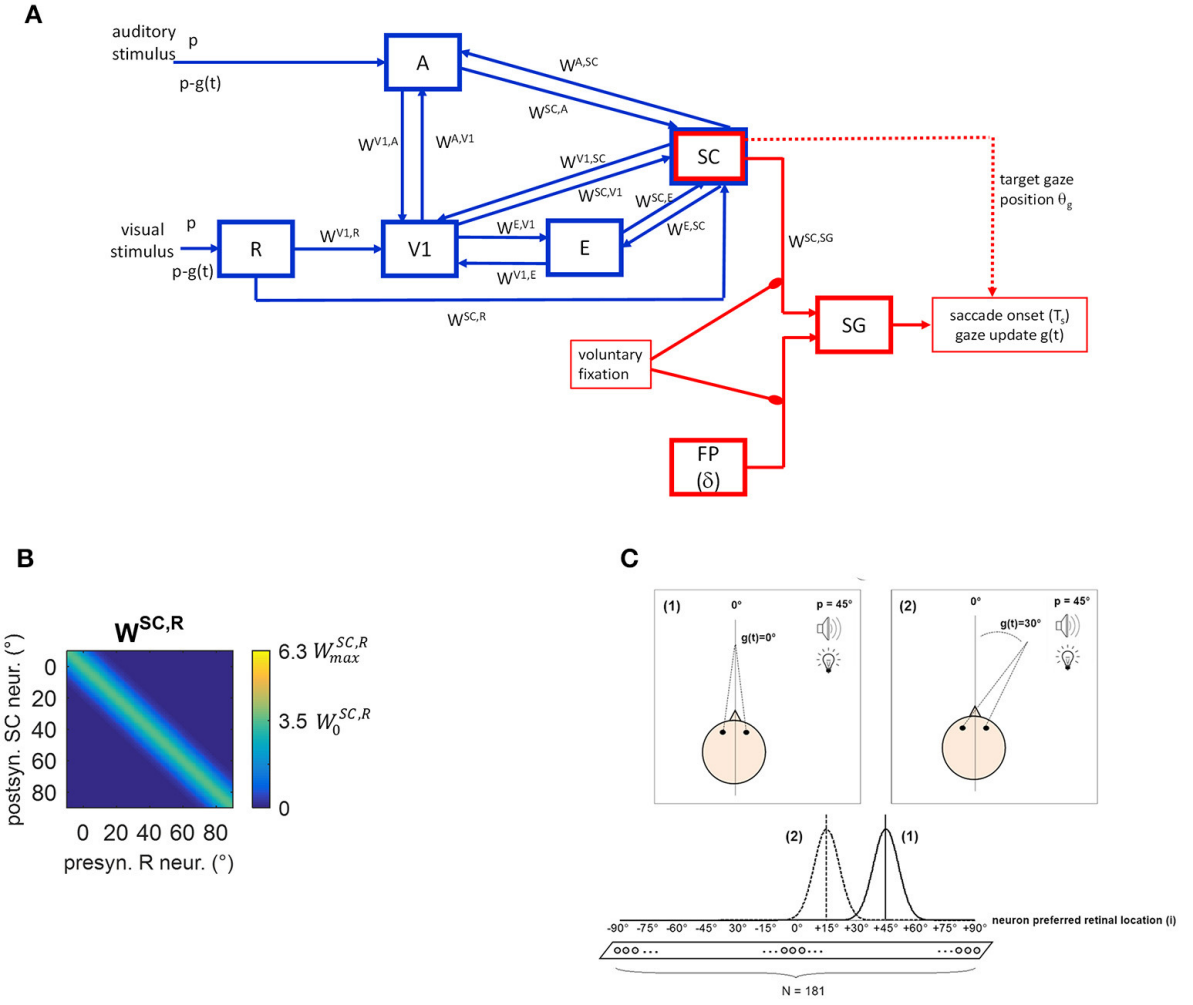
**(A)** Sketch of the neural network architecture. Blue blocks and lines represent the sensory module; red blocks and lines denote the oculomotor module. R, retina; V1, primary visual cortex; E, extrastriate visual cortex; SC, Superior Colliculus; A, auditory area; FP, saccade-related frontoparietal areas (δ denotes a pure delay); SG, Brainstem Saccade Generator. *g(t)* is the current gaze position (resulting from the oculomotor module); θ_*g*_ is the target gaze position decoded from the SC activity. *p* is the position of the external (visual or spatially coincident audiovisual) stimulus in head-centered coordinates, and *p-g(t)* is the stimulus position in retinotopic coordinates. *W*^*H, Q*^ denotes inter-area synapses from neurons in area *Q* to neurons in area *H*. **(B)** Exemplary pattern of basal (i.e., pre-training) inter-area synapses. Here, synapses *W*^*SC, R*^ from the retina to SC are depicted, limited to about one hemifield (−10° ÷ +90°) (the same pattern holds for the remaining not shown positions). x-axis reports the position (*j*, in deg) of the pre-synaptic neuron in area *R* and the y-axis the position (*i*, in deg) of the post-synaptic neuron in area *SC*. The color at each intersection (*j, i*) codes the strength of the synapse from the pre-synaptic neuron *j* in area *R* to the post-synaptic neuron *i* in *SC*. Similar patterns hold for all other inter-area synapses within the sensory module. Consistently with the following figures, scale color is between 0 and the maximum value reachable by training ($W_{max}^{SC,R}$). $W_{0}^{SC,R}$ denotes the central weight of the pre-training Gaussian pattern of the synapses. **(C)** Schematic picture of the eye-centered topological organization of neurons in each area. In case (1), the stimulus induces an activation bubble centered on the neuron with preferred retinal position = 45°, in a given area; in case (2), the stimulus induces an activation bubble centered on the neuron with preferred retinal position = 45°–30° = 15°.

The neural network is conceptually made up of two modules (Figure 1A). A sensory module (blue blocks and lines) includes cortical and subcortical (SC) neuronal areas devoted to the sensory representation of the external stimulation. An oculomotor module (red blocks and lines) can potentially react to the sensory neural representation, generating a saccade toward the external stimulation. The SC is involved in both modules.

Compared to our previous work (Magosso et al., 2016), the present neural network has been enriched by points of novelty. Indeed, the oculomotor model was previously absent and is implemented here to mimic saccade generation. Therefore, while in our previous study only condition of central fixation was simulated (*Fixed-Eyes Condition* with eye-centered and head-centered reference frames aligned), here gaze shift for stimulus foveation (without head movements) is simulated too (*Eye-Movements Condition*). Importantly, during an eye movement, the neural representation of the stimulus in the sensory network updates dynamically while the gaze shifts. To account for these aspects, here we distinguish between the eye-centered spatial coordinates (*i*) and the head-centered spatial coordinates (*h*), as they may not be aligned during a simulation. We denote with *g(t)* the gaze position (resulting from the oculomotor module) in head-centered coordinates, with 0° representing the central head orientation. Hence, at any time *t*, head-centered and eye-centered coordinates are related by the relationship *i* = *h* − *g(t)*, and the two reference frames coincide in case of central fixation. Moreover, while our previous study did not mimic synaptic training, here we assigned a priori basal values to the synaptic connections linking different neural areas, but they can change during training procedures via Hebbian learning rules.

In the following, we first provide a functional description of the sensory module (section The Sensory Module) and oculomotor module (section The Oculomotor Module) in their basal intact configuration mimicking a healthy subject. Then, network modifications to mimic hemianopic patients (section Simulation of Hemianopic Patients and Decoding of Visual Stimulus Detection), and the simulations performed to train and test the patients (section Simulation Schemes: Training Paradigms Implementing Synaptic Learning Rules and Testing Trials) are described.

### The sensory module

#### Description

This module was drawn from our previous paper (Magosso et al., 2016), maintaining architecture, equations and basal parameters (Table 1). Here, we present its qualitative description (while equations are provided in the Supplementary Material), and integrate additional specifications due to eye movement implementation.

**Table 1:** Basal values of network parameters and of synaptic learning rule parameters.

***External input***			
*p* depending on the trial		*V*_0_ = 20	σ_*V*_ = 2
		*A*_0_ = 17	σ_*A*_ = 32
*Gaussian noise on visual input* = 0 ± 0.1·*V*_0_		*Gaussian noise on auditory input* = 0 ± 0.1·*A*_0_	
***SENSORY MODULE***			
***Neuron input-output relationship***			
τ = 3 ms	φ = 12	ξ = 0.6	$y_{th}^{det}=$ 0.2 (*detection threshold* in V1 and E)
***Lateral synapses***			
*L*_*ex*0_ = 2	*L*_*in*0_ = 1.4 (areas *R, V1, E, A*)	σ_*ex*_ = 2	σ_*in*_ = 24 (areas *R, V1, E, A*)
$L_{ex0}^{SC}$ = 0	$L_{in0}^{SC}=$ 3		$\sigma_{in}^{SC}=$ 24
***Inter-Area Synapses (basal, i.e. pre-training, pattern)***			
***Amplitude (central weight)***		***Standard deviation***	
$W_{0}^{V1,R}$ = 5	$W_{0}^{SC,R}$ = 3.5^*^	σ^*V*1, *R*^ = 4	σ^*SC, R*^ = 8
$W_{0}^{SC,V1}$ = 0.5^*^	$W_{0}^{V1,SC}$ = 0.4	σ^*SC, V*1^ = 8	σ^*V*1, *SC*^ = 8
$W_{0}^{E,V1}$ = 3	$W_{0}^{V1,E}$ = 1	σ^*E, V*1^ = 6	σ^*V*1, *E*^ = 6
$W_{0}^{SC,E}$ = 0.5^*^	$W_{0}^{E,SC}$ = 0.75	σ^*SC, E*^ = 8	σ^*E, SC*^ = 8
$W_{0}^{SC,A}$ = 2.5^*^	$W_{0}^{A,SC}$ = 0.25	σ^*SC, A*^ = 16	σ^*A, SC*^ = 16
$W_{0}^{A,V1}$ = 0.4	$W_{0}^{V1,A}$ = 0.4	σ^*A, V*1^ = 8	σ^*V*1, *A*^ = 8
***OCULOMOTOR MODULE***			
***SG neuron and SC-SG connection***			
τ_*SG*_ = 70 ms	φ = 12	ξ = 0.6	$y_{th}^{SG}$ = 0.65 (*saccade threshold* in SG)
$W_{0}^{SG,SC\;}=1.1$	*T*_*s*_ (saccade onset)		
***Eye Movement control (empirical)***			
θ_*g*_ (gaze target decoded from SC at *T*_*s*_)		ν_*g*_ = 0.4°/ms (saccade velocity)	
***Synaptic Learning Rules***			
$W_{max}^{H,Q\;}=1.8\cdot W_{0}^{H,Q\;}\forall \;H,\;Q\;with\;H\neq SG$ γ_0_ = 8·10^−4^			
$W_{max}^{SG,SC\;}=3.6\cdot W_{0}^{SG,SC}$			
$y_{th}^{pre}=y_{th}^{post}$ = 0.2 (*learning threshold*) in areas R,E,V1,SC,SG			
$y_{th}^{pre}=y_{th}^{post}$ = 0.7 (*learning threshold*) in area A			

* *These parameters are slightly modified compared to our previous paper (Magosso et al., 2016), in order to increase the influence that the slight variability of SC activation (due to noisy stimuli) exerts over SRT variability (see Figures 3, 4). It is worth noticing that results presented in our previous paper are still valid under these modifications*.

Five areas of neurons are involved (Figure 1A). Three are devoted to the visual stimulus processing: the retina (R), the primary visual cortex (V1) and the extrastriate visual cortex (E). Area A is devoted to auditory stimulus processing. The area representing the Superior Colliculus (SC) is multisensory. Neurons in each area have their own preferred position in the external space and are topologically organized (Magosso et al., 2008, 2016), that is proximal neurons code for proximal spatial positions. Here, we assume that neurons have a retinotopic organization, coding spatial positions in eye-centered coordinates. This organization is justified in areas V1 and E, since striate and extrastriate visual areas are known to contain retinotopic maps (for a review Grill-Spector and Malach, 2004). As to SC, there is evidence that the multisensory SC layers receiving converging visual and auditory signals, are organized according to a motor-error map in eye-centered coordinates (Jay and Sparks, 1987b; May, 2006; Lee and Groh, 2012): that is, neurons in these layers encode gaze shift with a particular direction and amplitude for a given location on the map. On the contrary, brain areas involved in auditory localization are conventionally assumed to use a head-centered frame of reference derived from monaural and binaural cues. It is still uncertain which mechanisms and structures transform head-centered auditory space representation into eye-centered representation for coherent binding with visual information and for accessing the correct efferent zones in the SC. However, studies indicate that eye-position signals modulate auditory responses event at early stages in auditory processing (Groh et al., 2001), suggesting that the transformation from head- to eye-centered coordinates may occur gradually along the auditory pathway and possibly ending in the SC (Jay and Sparks, 1987b; May, 2006; Lee and Groh, 2012). Here, we made the simplified assumption that this transformation is completely accomplished at the level of area A. The latter, therefore, does not represent a specific auditory cortical area but is equivalent to several auditory processing stages that extract auditory stimulus position in eye-centered coordinates.

In line with our previous paper, we assume that from trial to trial the position at which the external stimulus (visual or auditory) is presented can vary only along the azimuth. Hence, neurons in each area are arranged along a monodimensional chain. Each area includes *N* = 181 neurons, with preferred retinal positions from −90° to + 90°, at a distance of 1° from each other, with 0° representing the current gaze position (i.e., the position of the fovea). In each area neurons are labeled by their preferred retinal position (*i* = −90°, −89°, …−1°, 0°, +1°, …, +89°, +90°). Of course, a single neuron in the model is not representative of a single biological cell but it represents an ensemble of cells functionally interconnected and sharing the same spatial properties.

Each neuron is described via an input-output relationship, including a sigmoidal function (ranging between 0 and 1, representing neuron's activation function) and a first-order dynamics mimicking the membrane time constant. Hence, neuron's activity (output) assumes value between 0 (silent neuron) and 1 (maximally activated neuron). The input to a neuron may comprehend a contribution due to the external stimulation (in areas directly receiving the external stimulus, i.e., R and A) and contributions due to synaptic connections.

In the following, the position *p* where the external stimulus is applied will be expressed in head-centered coordinates. The external visual and auditory stimuli are simulated as Gaussian functions of the difference between the stimulus position (re-computed in eye-centered coordinates) and neuron preferred position, spreading over a limited portion of space. Spatial extension was set larger for the auditory stimulus to account for the lower spatial resolution of the auditory receptors compared to the visual ones. The amplitude was set lower for the auditory stimulus (*A*_0_ = 17) than for the visual one (*V*_0_ = 20) to simulate lower saliency of the auditory stimulus. Indeed, in the present study, we focus on tasks where the visual stimulus is the target, and the auditory one is an accessory stimulus (see also section Simulation Schemes: Training Paradigms Implementing Synaptic Learning Rules and Testing Trials). Moreover, Gaussian noise with 0 mean and std = 10% of stimulus amplitude is superimposed on the external stimulus to introduce variability in neurons activity in response to external stimulation.

Two kinds of connections are implemented within the network. Lateral intra-area synapses connect neurons within the same area and realize near excitation and far inhibition (via Mexican-Hat disposition). Inter-area synapses (*W* in Figure 1A) are excitatory and connect neurons in different areas. In the following $W_{i,j}^{H,Q}$ will denote the synaptic weight from the pre-synaptic neuron *j* in area *Q* to the post-synaptic neuron *i* in area *H*. In the basal (pre-training) configuration, they are modeled as Gaussian functions of the distance between the preferred positions of the pre-synaptic and post-synaptic neurons. An exemplary pattern of basal inter-area synapses is shown in Figure 1B. However, inter-area synapses are subjected to synaptic plasticity (see section Training Paradigms and Learning Rules), hence their value and shape can change after training. We assumed that lateral synapses are not subject to training.

Due to the adopted architecture, an external stimulus applied at spatial position *p* expressed in head-centered coordinates, at any time *t*, will match the preferred location for neuron *i* = *p–g(t)* in the areas (see Figure 1C). Accordingly, in Eye-Movements Condition [see section Functioning of the Oculomotor Module (Intact Configuration)], each layer becomes a dynamic map of sensory activity: as gaze position changes, activity shifts to a new location corresponding to the new retinotopic position of the stimulus.

The pathways included in the module can be explained as follows. The external visual stimulus excites the retina (R), which sends visual information along two pathways. The pathway from R to V1 mimics the primary retino-geniculo-striate pathway; V1 is then reciprocally connected with extrastriate visual cortex (E). The pathway from R to SC mimics the secondary retino-collicular pathway, which sends direct ascending projections to the Colliculus. The SC also receives connections from the visual cortices V1 and E. The distinction among the three visual areas (R, V1, and E) is crucial to simulate hemianopic patients who are characterized by selective lesion to V1 but can still take advantage of the spared R-SC pathway. The auditory stimulus excites the auditory area A that sends auditory information to the SC. Besides these feedforward pathways, the network includes feedback mechanisms. One involves feedback synapses from the SC to areas A, V1, and E. The other involves reciprocal synapses between areas A and V1. Via these mechanisms, a stimulus in one modality may influence neural responses to a stimulus in the other modality, not only in the multisensory SC area but also in the respective unisensory areas. Our previous paper (Magosso et al., 2016) evidenced that the feedback synapses from the SC have a primary role in mediating audiovisual “online” effects in hemianopia.

#### Functioning of the sensory module (intact configuration)

**Figure 2:**
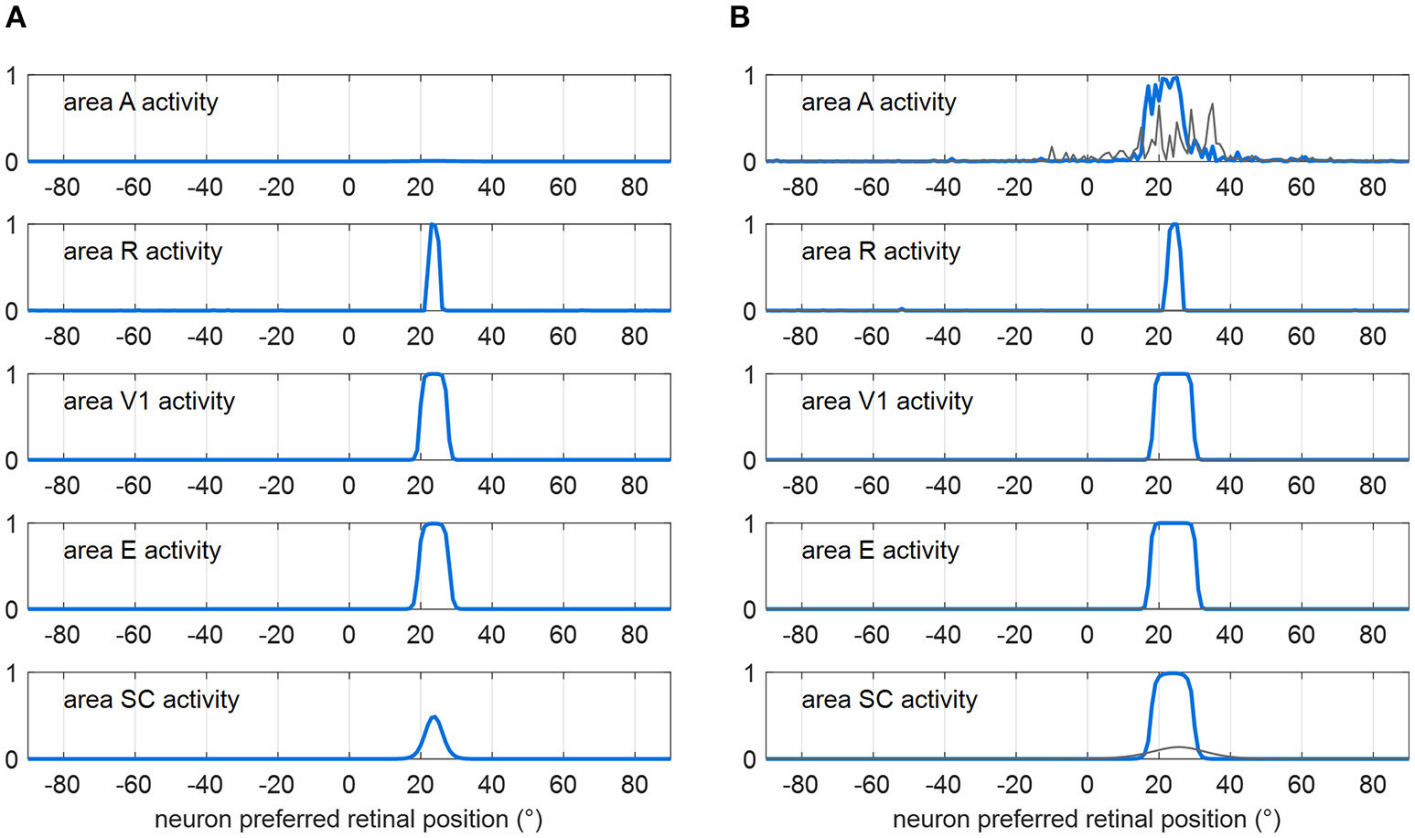
Exemplary functioning of the sensory module in intact configuration (gaze is at 0°, and the sensory module is intended disconnected from the oculomotor module). **(A)** Activation in all areas in response to a unisensory visual stimulus at *p* = 24°. Each plot shows the activity of all 181 neurons in the corresponding area. Neuron activity can assume value between 0 and 1 (maximum activity, see section Description). **(B)** Activation in all areas in response to a multisensory audiovisual stimulus at *p* = 24°. For comparison, activation in response to a unisensory auditory stimulus is shown by thin gray lines. The noise superimposed on the external stimulus produces a more irregular activation in area A then in R because of the larger Gaussian function simulating the auditory stimulus (see section Description and Table 1). In both panels **(A,B)**, the response is shown after the network has reached a steady-state activation profile in all areas.

Figure 2 shows how the sensory module works in response to a visual stimulus alone (Figure 2A) and to a multisensory audiovisual stimulus (Figure 2B), with basal parameter values and in intact condition. The visual stimulus activates all visual areas up to saturation, and the SC to a middle level. Under multisensory stimulation (although the auditory stimulus alone is little effective, see gray lines in Figure 2B), strong multisensory enhancement occurs in the SC that exhibits a wider bubble of activation and higher neuronal activity compared to unisensory visual stimulation.

### The oculomotor module

#### Description

The oculomotor module is devoted to initiation and driving of a saccade in response to the external stimulation. It concerns with reflexive saccades, elicited by the stimulus and generated to align the fovea with it. Frontoparietal (including Frontal Eye Fields, Supplementary Eye Fields, Posterior Parietal Cortex and Dorso-lateral Prefrontal Cortex) and subcortical (collicular) pathways are known to play a sophisticate parallel role in the initiation and control of saccades (Munoz, 2002; Sparks, 2002; Johnston and Everling, 2008; McDowell et al., 2008).

Here, we adopt an oversimplified structure, comprising only a few functional blocks whose interconnection is limited to recreate motor commands to brainstem and latency in saccade initiation (i.e., Saccade Reaction Time, SRT). Specifically, a single unit representing the brainstem saccade generator (SG) receives excitatory inputs from the SC and frontoparietal (FP) areas (Figure 1A); the FP block is modeled empirically and includes a pure delay δ (parameter values for this module are listed in Table 1).

As the other neuronal units in the network, the saccade generator unit (SG) filters its overall input via a sigmoidal function and a first-order dynamics to generate its output. We assumed that a saccade is initiated only when the SG output activity reaches a given threshold (saccade threshold). Hence

(1) $$\begin{array}{l} \tau_{SG}\frac{dy^{SG}\left(t\right)}{dt}=\;-y^{SG}\left(t\right)+F\left(u^{SG}\left(t\right)\right) \end{array}$$

(2) $$\begin{array}{l} F\left(u^{SG}\left(t\right)\right)=\frac{1}{1+\exp\left(-\left(u^{SG}\left(t\right)-\varphi \right)\cdot \xi \right)} \end{array}$$

(3) $$\begin{array}{l} Saccade\;is\;initiated\;at\;time\;T_{s}\;\Leftrightarrow \exists T_{s}{:y}^{SG}\left(T_{s}\right)=y_{th}^{SG} \end{array}$$

*u*^*SG*^(*t*) is the overall input to SG including the inputs from SC and FP (see Equation 4 below), and *y*^*SG*^(*t*) is SG output activity. *F*(·) is the sigmoidal activation function; its parameters are the same as for the other neurons. *T*_*s*_ is time of saccade onset, and $y_{th}^{SG}$ denotes the saccade threshold. The values for the time constant τ_*SG*_ (=70 ms) and saccade threshold $y_{th}^{SG}$ (=0.65) were assigned so that in any condition (especially during and after training when the circuit R-SC-SG is greatly reinforced) minimum saccade latency cannot decrease below ~75–80 ms which is the shortest saccade latency observed in humans (Bibi and Edelman, 2009; Knox and Wolohan, 2015), consistent with the neural delay of the shortest pathway from the retina to brainstem via the SC [(Boch et al., 1984), see also section The Oculomotor Module in the Discussion].

The overall input to SG, *u*^*SG*^(*t*), is computed as follows

(4) $$\begin{array}{l} u^{SG}\left(t\right)=\;u^{SG,SC}\left(t\right)+\;u^{SG,FP}\left(t\right)\\ =\overset{N}{\underset{j=1}{\sum }}W_{j}^{SG,SC}{\cdot \;y}_{j}^{SC}\left(t\right)\cdot \left(1-\alpha_{fix}\right)\\ +\;I_{0}^{SG,FP}\cdot \;r\left(t-\delta \right)\cdot \left(1-\alpha_{fix}\right) \end{array}$$

(5) $$r\left(t\right)=\{\begin{array}{l} 1,\;0\leq t\leq D\\ 0,\;otherwise\; \end{array}$$

(6) $$\alpha_{fix}=\{\begin{array}{l} \text{​​​​​​​​​​​​​}1,\;in\;Fixed-Eyes\;Condition\\ 0,\;in\;Eye-Movements\;Condition \end{array}$$

**Figure 3:**
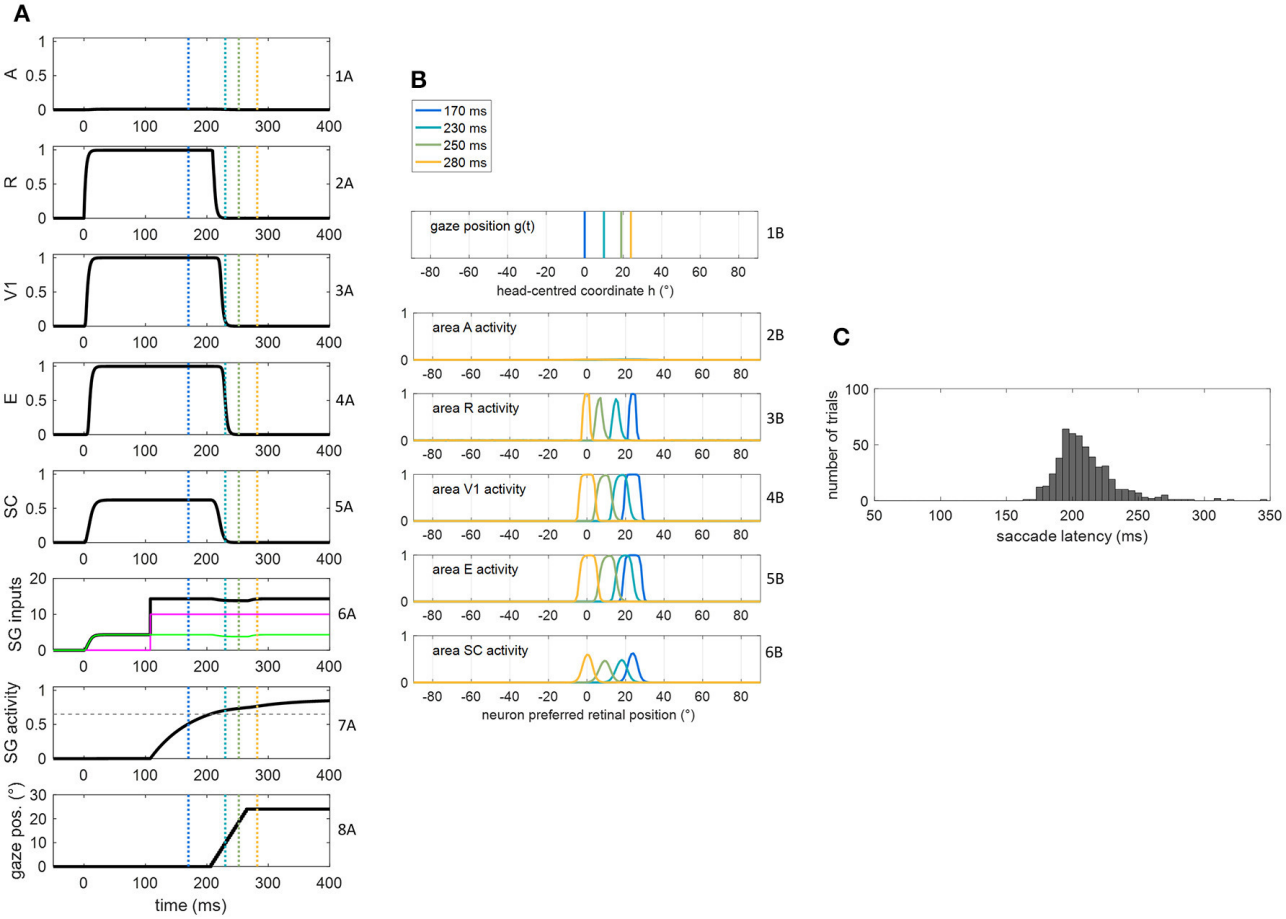
**(A,B)** Exemplary functioning of the oculomotor module, interconnected with the sensory module in the intact configuration, in a trial of unisensory visual stimulation applied at *p* = 24°. The stimulus lasts 400 ms starting from *t* = 0. The simulation starts with the gaze at central position 0°. Plots in panel **(A)** have the following meaning. Rows from 1A to 5A: temporal activity of the neuron having preferred retinal position 24°, in each area of the sensory module (activity of each neuron may range between 0 = silent neuron and 1 = maximally activated neuron). Row 6A: temporal profile of the inputs to SG (green line is SC input, magenta line is FP input, black line is the sum of the two inputs). Row 7A: temporal activity of the SG motor unit (activity of the neuron may range between 0 = silent neuron and 1 = maximally activated neuron); the dashed horizontal gray line indicates the saccade threshold ($y_{th}^{SG}$ = 0.65). Row 8A: gaze position along time, i.e., *g(t)*. Plots in panel **(B)** have the following meaning. Rows from 1B to 6B display the gaze position and overall activation in each area of the sensory module at different time instants (coded by different colors). The corresponding time instants are marked by colored vertical lines in the plots of **(A)**. **(C)** Saccade reaction time distribution obtained by performing 500 trials of unisensory visual stimulation, with each stimulus lasting 400 ms.

**Figure 4:**
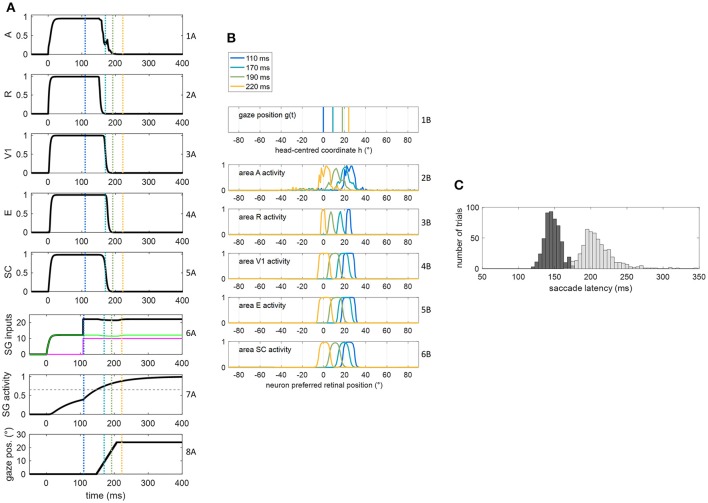
**(A,B)** Exemplary functioning of the oculomotor module, interconnected with the sensory module in intact configuration, in a trial of audiovisual stimulation applied at *p* = 24° and lasting 400 ms. The plots have the same meaning as Figure 3. **(C)** Saccade reaction time distribution obtained by performing 500 trials of audiovisual stimulation, with each stimulus lasting 400 ms (dark gray bars). For comparison, the light gray bars replicate SRT distribution in unisensory visual condition, i.e., the same as in Figure 3C.

The input from the SC (*u*^*SG, SC*^(*t*)) is computed via inter-area synapses $W_{j}^{SG,SC}$ projecting from the SC neurons to brainstem. In basal pre-training condition, synapses $W_{j}^{SG,SC}$ have a uniform value ($W_{j}^{SG,SC}=W_{o}^{SG,SC},\;\forall \;j\;\in SC$); however, as the other inter-area synapses, they are subjected to plasticity and can be modified during training (see section Training Paradigms and Learning Rules). The input from FP $\left(u^{SG,FP}\left(t\right)\right)=I_{0}^{SG,FP}\cdot r\left(t-\delta \right)$ has been modeled empirically, as a constant input having the same duration *D* as the stimulus, and delayed from the stimulus onset (*t* = 0) by a given amount (δ = 100 ms ± 10 ms, normally distributed), in line with visual response latencies in these structures (Boch et al., 1984; Lamme and Roelfsema, 2000). Because of this delay, the SC input anticipates the FP input to SG [see section Functioning of the Oculomotor Module (intact configuration) and Figures 3, 4]. The value of the FP input ($I_{0}^{SG,FP}=10$) and the basal value of the synapses from SC to SG ($W_{0}^{SG,SC}=1.1$) were set to satisfy the following requirements. (i) In basal (pre-training) condition, only the combination of the two inputs (and neither input alone) is highly likely to trigger a saccade. This agrees with data showing that lesions in either saccade-related frontoparietal areas or SC induce saccade deficits (Johnston and Everling, 2008; McDowell et al., 2008). (ii) Saccade reaction time under unimodal visual stimulation (when the SC exhibits a lower activation, see Figure 2A), is ≈200 ms and decreases by ≈50–60 ms under audiovisual stimulation (when the SC exhibits a stronger activation, see Figure 2B), in line with human data [(Nozawa et al., 1994; Colonius and Arndt, 2001; Sparks, 2002; Bargary et al., 2017); see section Functioning of the Oculomotor Module (Intact Configuration) and Figures 3, 4].

α_*fix*_ in Equations (4) and (6) is a flag used to simulate both Fixed-Eyes Condition (in which the subject keeps central fixation) and Eye-Movements Condition (in which the subject is free to generate a saccade toward the stimulus). Specifically, α_*fix*_ avoids saccade generation under voluntary fixation, inhibiting the inputs to the SG in Fixed-Eyes Condition. Notably, even in Eye-Movements Condition, no oculomotor response may occur in reaction to the external stimulation if the latter does not produce suprathreshold SG activation (e.g., when the combination of the inputs from SC and FP is too low and/or one input is lacking).

Finally, in case a saccade is initiated (i.e., in case *T*_*s*_ exists), the eye movement is driven empirically. SC activation at the time of saccade onset (*T*_*s*_) is read out according to a winner-takes-all rule: the position coded by the maximally activated *SC* neuron at that time is assumed to signal the target gaze displacement (θ_*g*_). Then, for *t* > *T*_*s*_, gaze position *g*(*t*) is updated at a constant rate, as long as its value is different from the target value. Hence

(7) $$g\left(t\right)\;=\{\begin{array}{c} 0,\;\begin{array}{c} \;\forall t:0\leq t\leq T_{s},\;if\;T_{s}\;exists\\ \;\forall t,\;if\;T_{s}\;does\;not\;exist \end{array}\\ sign\left(\theta_{g}-g\left(t-\Delta t\right)\right)\cdot v_{g}\cdot \left(t-T_{s}\right),\;\forall t:\left(t>T_{s}\right)\wedge \left(g\left(t-\Delta t\right)\neq \theta_{g}\right),\;if\;T_{s}\;exists \end{array}$$

where

(8) $$\theta_{g}=arg\underset{i}{\max}\left\{y_{i}^{SC}\left(T_{s}\right)\right\},\;if\;T_{s}\;exists$$

In Equation (7), Δ*t* is the simulation time step (=0.1 ms) and *v*_*g*_ is the saccade velocity set equal to 0.4°/ms, regardless of saccade amplitude, in agreement with velocity average values (Garbutt et al., 2001; Sparks, 2002).

#### Functioning of the oculomotor module (intact configuration)

Figures 3, 4 show the exemplary functioning of the oculomotor module, interconnected with the sensory module, and evidence how the parameter adjustment in this module recreates SRT data in line with *in vivo* studies.

Figures 3A,B show the dynamic operation of the network in response to a visual stimulus (lasting 400 ms) applied at *p* = 24°. The trial starts with the gaze at central position = 0°. Following stimulus appearance (at *t* = 0), neurons in visual and SC areas coding position p activate rapidly (rows from 2A to 5A), and the input from SC to SG assumes a non-null value (green line in row 6A). However, before the FP input to SG becomes operative (magenta line in row 6A), the SC input alone is ineffective, keeping SG unit silent (row 7A). At the arrival of FP input (*t* = 108 ms), the accumulation of the two inputs gradually rises SG activity toward the saccade threshold (0.65) that is reached at t = 206 ms (hence SRT = 206 ms). Starting from this time, the gaze position [*g(t)*] moves to reach the target position (=24°, decoded from SC peak activity at saccade onset) at a constant rate (row 8A). Due to the retinotopic organization of the neuronal areas, while gaze position moves from 0° to 24° (row 1B), activity in each area moves in the opposite direction compared to the gaze, continuously matching the changing in the retinotopic position of the stimulus (rows from 2B to 6B). Accordingly, neurons coding the initial retinotopic position of the stimulus rapidly becomes inactive following eye movement onset (rows 2A ÷ 5A). As activity in SC shifts, SC input to SG is maintained (since all SC neurons project to SG). Here, the stimulus lasts sufficiently for the gaze to reach the target (yellow lines in Figure 3B). Saccade latency distribution obtained in 500 visual trials (Figure 3C) shows a peak at ~200 ms and values mainly within 170–230 ms, replicating *in vivo* data of regular saccade latencies to unisensory visual stimuli (Colonius and Arndt, 2001; Sparks, 2002; Bargary et al., 2017). Variability in SRT does not arise only from variability in the FP delay but also from variability in SC activation due to the noisy sensory input.

The dynamic operation of the network in response to a multisensory audiovisual stimulus (lasting 400 ms) is reported in Figures 4A,B. Thanks to the multisensory enhancement in the SC, the earlier SC input to SG (green line in row 6A) moves the SG unit out of its silent state (up to about 0.4, row 7A). Hence, shortly after the arrival of the additional FP input (at *t* = 108 ms), the SG unit reaches the saccade threshold at *t* = 147 ms. In audiovisual condition, also activity in area A shifts (row 2B) while the gaze shifts. Saccade latency distribution obtained by performing 500 audiovisual trials (Figure 4C) show that both the average value and standard deviation decrease in case of bimodal stimulation (147 ± 11 ms) compared to unimodal stimulation (207 ± 30 ms), recreating *in vivo* findings (Nozawa et al., 1994; Colonius and Arndt, 2001). These same studies mainly ascribe reduced saccade latency in audiovisual condition to SC multisensory convergence, as the model postulates, too.

It is worth noticing (as relevant for subsequent considerations, see section Simulation Schemes: Training Paradigms Implementing Synaptic Learning Rules and Testing Trials) that, according to the model, in the early ~100 ms of stimulus presentation, only the input from the SC acts on the SG unit (because of the FP input delay). Basal parameterization of the oculomotor model (in particular synapses from SC to SG) are not likely to produce short latency (<100 ms) saccades, especially in unimodal condition, despite the fast sensory response in SC. This basal setting of the model agrees with studies on SRT in healthy human and non-human primates indicating that express saccades (i.e., saccades that can reach latency below 100 ms, as low 75–80 ms) are almost absent in basal condition and they are more likely to occur after individuals have had repeated trainings in specific visual paradigms (Fischer et al., 1984; Fischer and Ramsperger, 1986; Bibi and Edelman, 2009).

### Simulation of hemianopic patients and decoding of visual stimulus detection

The previous sections describe the intact network simulating a healthy subject. Hemianopia was mimicked by damaging the V1 area unilaterally, in the region corresponding to the retinotopic positions from +1° to +90°. Specifically, we simulated twenty different hemianopic patients, by randomly damaging (i.e., silencing) at least 85% of V1 neurons within this region (see (Magosso et al., 2016) for a detailed description of the procedure). Here, we adopted the same twenty simulated patients used in our previous paper (Table 2).

**Table 2:** Hemianopic patients simulated with the network.

**Patient**	**Number (*n*) of silenced *V1* neurons in the damaged hemifield**	**Preferred retinal positions of the spared *V1* neurons in the damaged hemifield**
1	*n* = 89	43°
2	*n* = 84	12° 19° 25° 66° 73° 86°
3	*n* = 82	33° 34° 39° 50° 51° 63° 64° 90
4	*n* = 87	56° 81° 82°
5	*n* = 88	41° 71°
6	*n* = 80	1° 27° 30° 38° 41° 55° 56° 59° 60° 88°
7	*n* = 87	7° 25° 56°
8	*n* = 81	10° 19° 26° 28° 47° 50° 77° 86° 88°
9	*n* = 85	8° 22° 27° 61° 76°
10	*n* = 87	42° 46° 49°
11	*n* = 76	1° 2° 20° 21° 24° 25° 31° 46° 50° 55° 63° 74° 77° 86°
12	*n* = 86	17° 25° 34° 75°
13	*n* = 79	5° 9° 11° 32° 33° 43° 45° 47° 49° 82° 86°
14	*n* = 79	8° 9° 10° 18° 23° 36° 48° 67° 68° 74° 75°
15	*n* = 80	12° 24° 28° 42° 50° 55° 65° 85° 86° 87°
16	*n* = 77	5° 17° 25° 35° 40° 46° 59° 61° 66° 67° 73° 83° 89°
17	*n* = 80	12° 51° 52° 55° 70° 71° 74° 79° 81° 84°
18	*n* = 89	29°
19	*n* = 77	5° 6° 9° 11° 20° 26° 34° 51° 56° 57° 68° 82° 85°
20	*n* = 76	3° 11° 17° 26° 31° 32° 36° 38° 44° 46° 52° 56° 58° 71°

Since the presence of spared V1 neurons is of relevance (see section Results and Discussion), some specifications are useful. Of course, a spared V1 neuron does not represent a single cell, rather a small spot of survived V1 tissue at a given retinotopic position. For a given position *p*, we say that a cluster of spared V1 neurons exists around that position if at least 2 survived V1 neurons are present within the range *p* − 4°÷ *p* + 4°, and the number of spared V1 neurons in this range defines the cluster's size around that position (Magosso et al., 2016). For example a 3-neuron cluster around *p* = 40°, means that three spared V1 neurons are present in the range 36° ÷ 44°.

Finally, we assumed that a visual stimulus is consciously detected only if, at some time *t* during the presentation of the stimulus, neuron activity in both areas V1 and E overcomes a given threshold ($y_{th}^{dect}$ = 0.2, detection threshold) (Magosso et al., 2016). Notably, here visual awareness may emerge during an oculomotor response (after training), if the eye movement moves the visual stimulus to regions where visual function is intact or has been restored (see section Results).

### Simulation schemes: training paradigms implementing synaptic learning rules and testing trials

The simulation trials (both training and testing trials) performed in the hemianopic patients share the following characteristics, to resemble the trials used in real patients (Bolognini et al., 2005; Passamonti et al., 2009; Tinelli et al., 2015; Grasso et al., 2016).

- Each trial involves the application of a visual stimulus or audiovisual spatiotemporally coincident stimulus, applied at *t* = 0 and lasting 100 ms. The stimulus can be presented at different eccentricities along the azimuth, within the blind hemifield.
- In each trial, the input from FP to SG is excluded. This exclusion is justified since only 100 ms stimuli are used in these simulations [see point (i) above], in agreement with studies on hemianopic patients, and—according to neurophysiological data of signal propagation time in frontoparietal structures (section The Oculomotor Module)—FP input has no or only a minor role due to its delay, in case of such a short stimulation. However, the development of a basal oculomotor module including the FP input is relevant. Indeed, in this way we provide an intact reference model that justifies the parameters of the collicular sensory-motor link used in pre-training condition and in synaptic learning in the simulated patients.
- Each trial starts with the simulated patient fixating centrally and can be performed in two different eye conditions: *Fixed-Eyes Condition*, in which central fixation is hold, inhibiting any oculomotor response; *Eye-Movements Condition*, in which a saccade can be produced, shifting the gaze from the central fixation point toward the stimulus.
- The visual stimulus (in unisensory and multisensory stimulations) represents the target, while the auditory stimulus is an accessory stimulus (in real studies, indeed, patients were instructed to detect the presence of the visual target, ignoring the auditory stimulus).

#### Training paradigms and learning rules

Each simulated hemianiopic patient was subjected to training paradigms, consisting of repeated stimulation trials of the blind hemifield at four different positions (8°, 24°, 40°, 56°). Three training paradigms were simulated.

*Training paradigm A* (Audiovisual training in Eye-Movements Condition)—In each trial, a spatiotemporally coincident audiovisual stimulation (100 ms) was applied and the oculomotor response was allowed.

*Training paradigm B* (Audiovisual training in Fixed-Eyes Condition)—In each trial, a spatiotemporally coincident audiovisual stimulation (100 ms) was applied and the oculomotor response was not allowed (inputs to SG were inhibited and SG unit remained silent).

*Training paradigm C* (Visual training in Eye-Movements Condition)—In each trial, a unimodal visual stimulation (100 ms) was applied and the oculomotor response was allowed.

For each paradigm, a full training session comprehended 300 stimulation trials, 75 trials per position. The four positions were stimulated in a random sequence during the session. Each training trial began at stimulus appearance (*t* = 0). After an initial interval was elapsed (settling time *T*_*off*_ = 65 ms), the training of inter-area synapses (*W*) started. The training trial continued until the removal of the stimulus (i.e., until *t* = 100 ms), in case visual detection did not occur during this time. In case at any time *t* between *T*_*off*_ and 100 *ms* the visual stimulus was detected, the trial was interrupted 10 ms after detection (or at *t* = 100 ms if detection occurs at a time *t* > 90 ms). This mimics *in vivo* studies where patients were asked to push a button as soon as the visual stimulus was detected (trial interruption mimics subject's engagement in pushing button). Synaptic learning in one trial started from the final synaptic configuration reached at the end of the previous trial.

We assumed biologically plausible learning rules to implement the training phase (Dayan and Abbott, 2001). We adopted rules already used to investigate other phenomena of multisensory plasticity in our previous studies (Magosso et al., 2012, 2013). The inter-area synapses within the sensory module modify according to a classic potentiation Hebbian rule with pre-synaptic and post-synaptic thresholding: the synaptic weight $W_{ij}^{H,Q}$ increases, up to a maximum saturation value $W_{\text{max}}^{H,Q}$, only if both pre-synaptic and post-synaptic neuron activities are above a given threshold ($y_{pre}^{Q}$ and $y_{post}^{H}$, respectively, named learning thresholds). Furthermore, these inter-area synapses are subjected to a normalization rule: the sum of all synaptic weights entering a given neuron in a generic area H (=A, V1, E, SC) remains constant. According to this rule, the increase of some synapses occurs at expense of other synapses. This helps to keep stability in the network, avoiding an uncontrolled propagation of excitation among areas during training, due to the several recurrent excitatory synapses in the sensory module. Thus, we have

(9) $$\begin{array}{l} \Delta W_{ij}^{H,Q}\left(t\right)=\gamma_{ij}^{H,Q}\left(t\right)\cdot {\left[y_{i}^{H}\left(t\right)-y_{post}^{H}\right]}^{+}\cdot {\left[y_{j}^{Q}\left(t\right)-y_{pre}^{Q}\right]}^{+} \end{array}$$

(10) $$\begin{array}{l} \gamma_{ij}^{H,Q}\left(t\right)=\;\gamma_{0}\cdot \left(W_{max}^{H,Q}-W_{ij}^{H,Q}\left(t\right)\right) \end{array}$$

(11) $$\begin{array}{l} W_{ij}^{H,Q}\left(t+\Delta t\right)=\frac{W_{ij}^{H,Q}\left(t\right)+\Delta W_{ij}^{H,Q}\left(t\right)}{\sum_{Q}\sum_{j}\left(W_{ij}^{H,Q}\left(t\right)+\Delta W_{ij}^{H,Q}\left(t\right)\right)}\\ \cdot \sum_{Q}\sum_{j}W_{ij}^{H,Q}\left(0\right)\;\forall H,Q=A,R,V1,E,SC\;H\neq R \end{array}$$

$\Delta W_{ij}^{H,Q}$ in Equation (9) denotes the change in the synaptic weight due to the potentiation rule. [ ]^+^ denotes the function positive part (i.e., [*x*]^+^ = *x* if *x* ≥ 0, while [*x*]^+^ = 0 if *x* < 0). Equation (10) implements maximum saturation by decreasing the current learning rule $\gamma_{ij}^{H,Q}\left(t\right)$ as the synaptic weight increases, and $\gamma_{0}\cdot W_{\text{max}}^{H,Q}$ is the maximum learning rate. Equation (11) implements the normalization rule. $W_{ij}^{H,Q}\left(0\right)$ denotes synaptic weight in the basal (pre-training) configuration; the outer sum at the denominator and numerator extends to all areas *Q* sending synapses to area *H*, and the inner sum extends to all neurons *j* within each area *Q*.

The inter-area synapses in the oculomotor module (${W_{j}}^{SG,SC}$) obey the pre-synaptic thresholding potentiation rule with upper saturation. Hence

(12) $$\begin{array}{l} \Delta W_{j}^{SG,SC}\left(t\right)=\gamma_{j}^{SG,SC}\left(t\right)\cdot y^{SG}\left(t\right)\cdot {\left[y_{j}^{SC}\left(t\right)-y_{pre}^{SC}\right]}^{+} \end{array}$$

(13) $$\begin{array}{l} \gamma_{j}^{SG,SC}\left(t\right)=\;\gamma_{0}\cdot \left(W_{max}^{SG,SC}-W_{j}^{SG,SC}\left(t\right)\right) \end{array}$$

(14) $$\begin{array}{l} W_{j}^{SG,SC}\left(t+\Delta t\right)=W_{j}^{SG,SC}\left(t\right)+\Delta W_{j}^{SG,SC}\left(t\right) \end{array}$$

where the meaning of the symbols is the same as in Equations (9–10).

The value of parameter γ_0_ was set sufficiently low to ensure a gradual updating of synapses during training. Maximum saturation value $W_{max}^{H,Q}$ for all synapses $W_{ij}^{H,Q}$ in the sensory module was set at 180% their basal central weight ($W_{0}^{H,Q}$). Maximum saturation value $W_{max}^{SG,SC}$ ($=3.6\cdot W_{0}^{SG,SC}$) for synapses $W_{j}^{SG,SC}$ was tuned so that in unisensory condition the SC alone can provide an input to SG almost equivalent to the sum of SC and FP inputs (when both operative) in the pre-training configuration. Neurons in all areas but area A were given the same values for pre-synaptic and post-synaptic thresholds (=0.2). For neurons in area A, pre-synaptic and post-synaptic thresholds were set at a higher level (=0.7) to mimic reduced attention to the auditory modality (as only the visual stimulus represented the target). The overall number of training trials (300) has been chosen since adding further trials does not provide further appreciable modifications in the synapses and in the post-training performances.

#### Testing trials

Before and after each training paradigm, each simulated patient was subjected to a testing session to assess the effect of training on visual detection performance. Each testing trial involves unisensory visual stimulation (100 ms). Two visual tests were considered, in agreement with (Bolognini et al., 2005).

*Visual Test 1* consisted in trials that visually stimulated the same spatial positions used during training (8°, 24°, 40°, 56°); 8 trials were repeated for each spatial position.

*Visual Test 2* consisted in trials that visually stimulated a dense array of positions in the hemianopic field (from 4° to 60° with 2° step); 2 trials were performed for each spatial position.

Each visual test was performed both in *Fixed-Eyes Condition* and in *Eye-Movements Condition* and visual detection accuracy (percentage of detected visual stimuli) was computed in each condition and averaged on all simulated patients.

## Results

Results obtained in the simulated patients (see patient description in section Simulation of Hemianopic Patients and Decoding of Visual Stimulus Detection), before and after training, are presented. All simulations are performed using stimuli lasting 100 ms (as specified in section Simulation Schemes: Training Paradigms Implementing Synaptic Learning Rules and Testing Trials).

### Pre-training functioning

**Figure 5:**
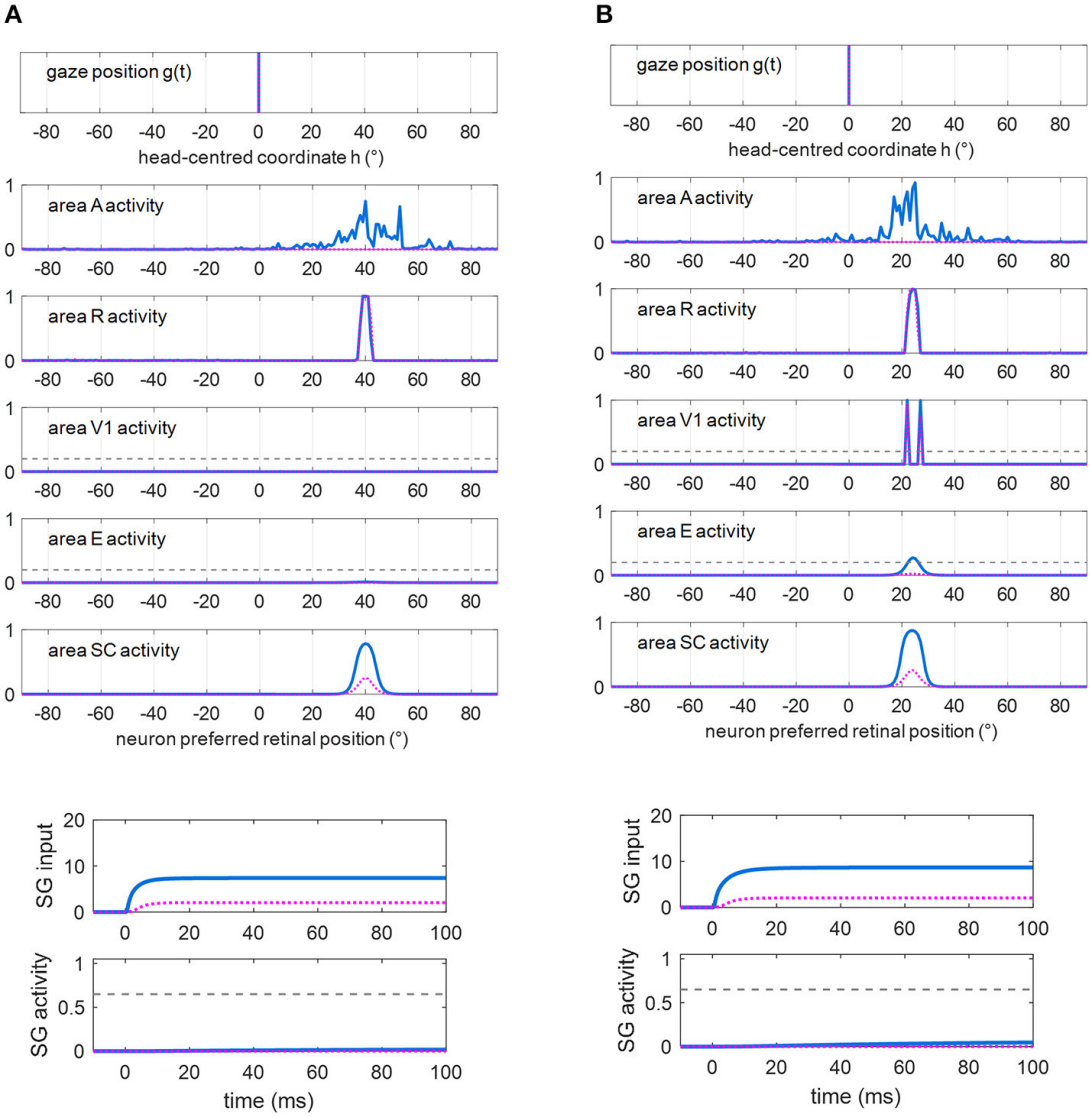
**(A)** Exemplary response of the network in the simulated patient #9 to a visual stimulus (dotted purple lines) and to an audiovisual stimulus (continuous blue lines) lasting 100 ms, applied at *p* = 40°. Around this position, no spared V1 neuron is present. The overall activation profile in areas A, R, V1, E, SC is shown at the end of the simulation (*t* = 100 ms); in area V1 and E, the horizontal gray dashed line indicates the detection threshold ($y_{th}^{det}$ = 0.2). The input to SG (that coincides with the input from SC), and SG activity is displayed as a function of time; in the latter plot, the horizontal gray dashed line indicates the saccade threshold ($y_{th}^{SG}$ = 0.65). **(B)** Exemplary response of the network in the simulated hemianopic patient #9 to a visual stimulus (dotted purple lines) and to an audiovisual stimulus (continuous blue lines) lasting 100 ms, applied at *p* = 24°. Around that position, two spared V1 neurons (2-neuron cluster) are present. The meaning is the same as in **(A)**. In both panels **(A,B)**, SC input to SG is insufficient to trigger SG activity even in case of audiovisual stimulation.

Figures 5A,B show exemplary responses to a visual alone and to a multisensory audiovisual stimulation in two representative cases corresponding to no spared V1 neurons (Figure 5A) and 2-neuron spared cluster (Figure 5B) around the stimulus position (simulations are drawn from patient #9). The simulations are performed in Eye-Movements Condition; however, since FP input to SG is ineffective (see section Simulation Schemes: Training Paradigms Implementing Synaptic Learning Rules and Testing Trials), the stimulation is unable to produce an oculomotor response via the SC input alone and the gaze remains at central position. In case (A), the visual stimulus activates only the retina, which produces a slight activity in the SC; the visual stimulus is not detected (areas V1 and E remain silent). In case (B), the visual stimulus produces activation of the retina, slight activation of the SC and also activation of the spared V1 neurons; the latter, however, do not provide sufficient input to activate area E and the stimulus remains undetected (suprathreshold activity in both areas V1 and E are required for stimulus detection, see section Simulation of Hemianopic Patients and Decoding of Visual Stimulus Detection). In both cases, adding an auditory stimulus elicits a strong multisensory enhancement in the SC. Notably, in case (B), the enhanced feedback from the SC to area E adds to the feedforward input from the spared V1 neurons, inducing a suprathreshold activity in region E and stimulus detection. In our previous paper (Magosso et al., 2016), we made an extensive analysis showing that at least a spared cluster (i.e., 2 spared V1 neurons) is required for visual detection to be triggered (in Fixed-Eyes condition) by an accessory auditory stimulus. That is, the enhanced multisensory feedback from SC can induce suprathreshold E activation only in presence of a minimum level of feedforward input from V1 to E, which is not provided by an isolated spared neuron (and is null in case of no spared V1 neuron). Finally, in both cases, multisensory enhancement in SC augments the input from SC to SG (see bottom plots), compared to unimodal visual condition, although insufficient to trigger the SG unit.

Stimulations applied in Fixed-Eyes Condition produce same patterns of activity in all areas of the sensory module, but the input to SG is inhibited by voluntary fixation.

### Training A (audiovisual stimulation in eye-movements condition) and post-training performances

**Figure 6:**
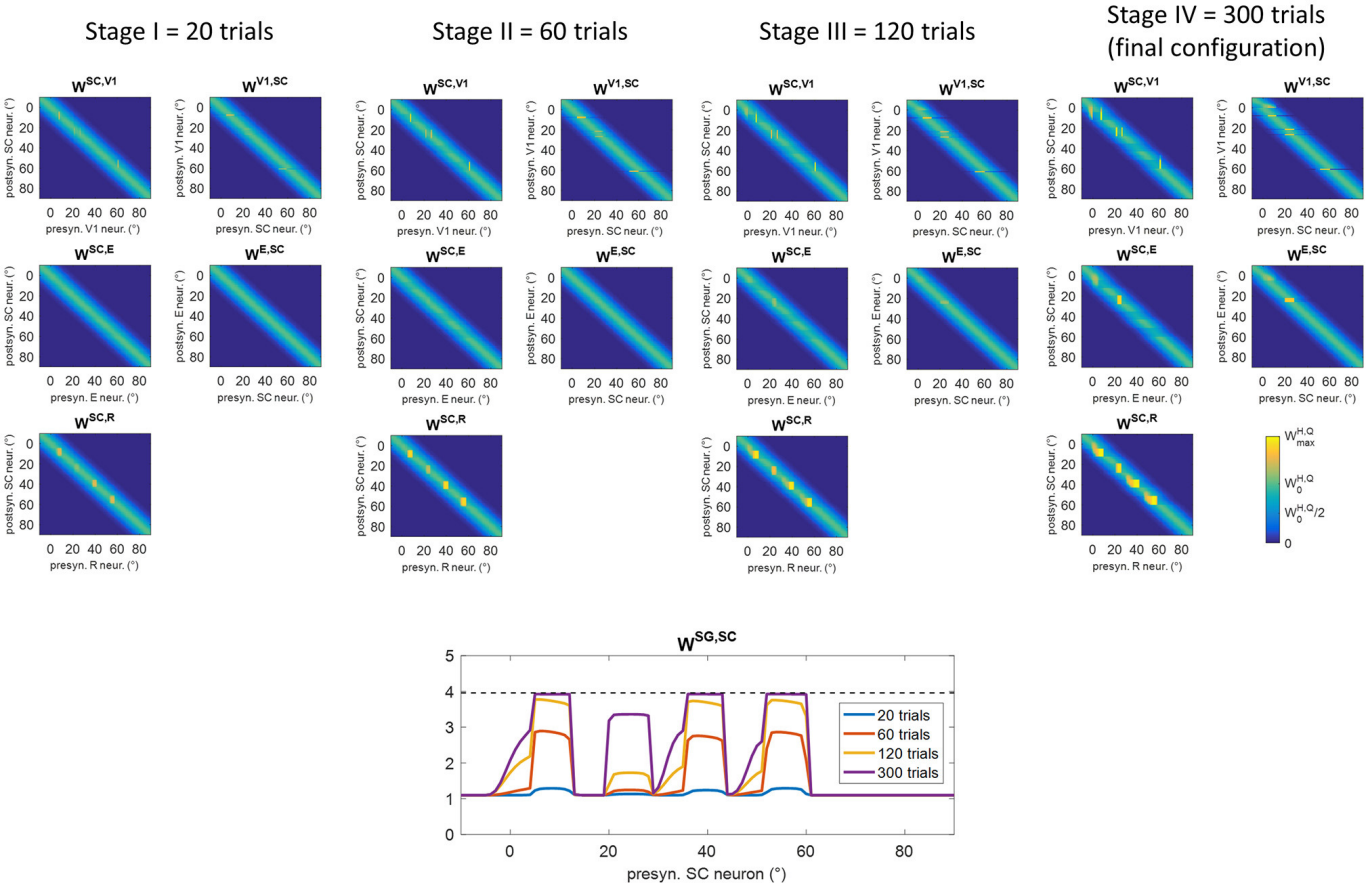
Pattern of inter-area synapses for patient #9 at four different stages of training A (after 20 training trials, 60 training trials, 120 training trials, 300 training trials corresponding to the end of training). At each stage, the four positions (8°, 24°, 40°, 56°) had received the same number of stimulations. For sake of space, only the most significant synapses are reported including synapses from the retina to SC (*W*^*SC, R*^), reciprocal synapses between SC and cortical visual areas V1 and E (*W*^*SC, V*1^, *W*^*V*1, *SC*^, *W*^*SC, E*^, *W*^*E, SC*^) and synapses from SC to SG (*W*^*SG, SC*^, bottom plot). Each color map has the same meaning as in Figure 1B, where x-axis denotes the position (*j*) of the pre-synaptic neuron in area *Q*, y-axis denote the position (*i*) of the post-synaptic neuron in area H, and the color value at the intersection (*j,i*) indicates the strength of the synapse $W_{ij}^{H,Q}$. In each color map, the color scale ranges between 0 and the maximum value $W_{max}^{H,Q}$ (Table 1) for the represented synapses. $W_{0}^{H,Q}$ is the central weight of the pre-training Gaussian pattern. The bottom plot, displaying synapses *W*^*SG, SC*^ at different stages, reports the position (*j*) of the SC presynaptic neuron on the x-axis (in the range −10° to 90°) and the value of the corresponding synapse ${W_{j}}^{SG,SC}$ on the y-axis. The dashed black line marks the value $W_{max}^{SG,SC}$. At the first stages of training (Stage I and II), synapses interconnecting active neurons around the stimulated positions start to reinforce. At the end of Stage II, synapses *W*^*SG, SC*^ at the stimulated positions become strong enough to elicit an oculomotor response rightward (so that an activation wave moves leftward). Hence, synaptic reinforcement extends leftward (Stages III and IV) but only to a limited extent as gaze moves only by a few degrees before stimulus removal, i.e., before the end of the trial. At position 24°, leftward extension of synaptic reinforcement does not occur as the stimulus is perceived before oculomotor response is triggered. At this position also reciprocal synapses *W*^*SC, E*^ and *W*^*E, SC*^ reinforce too. Modifications of synapses *W*^*SC, V*1^, *W*^*V*1, *SC*^, *W*^*SC, E*^, *W*^*E, SC*^around 0° (see Stage IV) arise from the activation produced in these areas when the stimulus reaches the fovea and the intact side.

Figure 6 shows changes in inter-area synapses at different stages during training A in an exemplary patient (patient #9, the same as in Figure 5). Among the four stimulated positions (8°, 24°, 40°, 56°), the patient exhibits a spared cluster around position 24°. Only changes in the most significant synapses are reported (see Figure S1 for changes in all inter-area synapses). The changes can be explained as follows. At the beginning of training (Stages I and II), oculomotor responses are not elicited as SC input to SG is still insufficient, and inter-area synapses start to reinforce around the stimulated positions. Reinforcement concerns synapses (*W*^*SC, R*^) connecting the retina to the bubble of SC neurons activated by the audiovisual stimulus (see for example Figure 5 blue lines), and reciprocal synapses between V1 and SC (*W*^*SC, V*1^, *W*^*V*1, *SC*^) at the positions of spared V1 neurons (position 24° in this case, see Figure 5B blue lines). Concerning synapses from SC to SG (*W*^*SG, SC*^), in the very early stage (Stage I) they exhibit very little reinforcement (blue line in the bottom panel of Figure 6), being the input from SC still insufficient to trigger SG activity. As learning proceeds (Stage II) and synapses entering SC (especially *W*^*SC, R*^) reinforce, the increased SC activity triggers SG neuron at a sufficient level to speed up the reinforcement of synapses *W*^*SG, SC*^ (red line in bottom panel of Figure 6). At the end of Stage II, synapses *W*^*SG, SC*^ have become strong enough to elicit an oculomotor response toward the stimulus.

**Figure 7:**
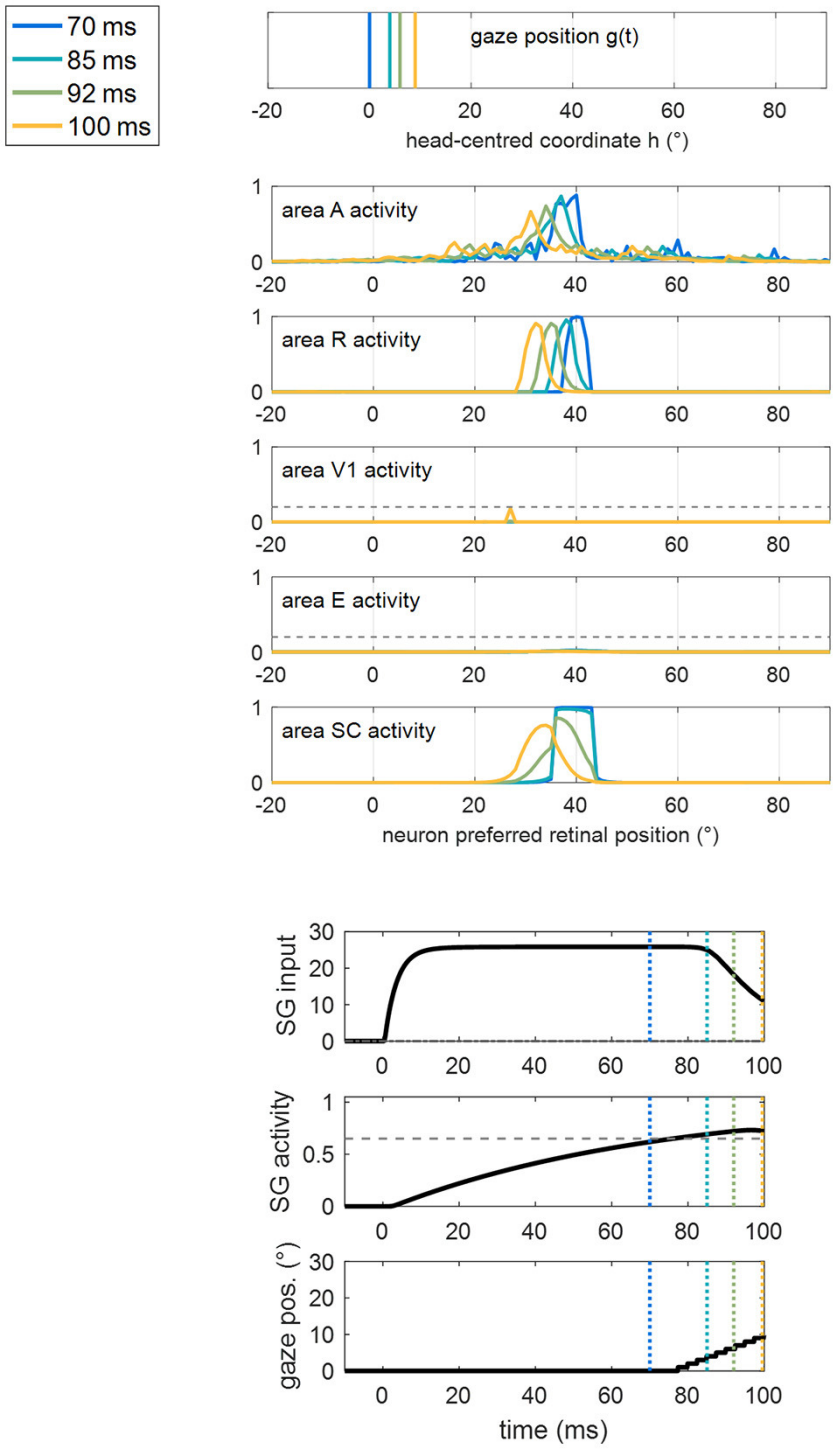
Example of the network response in patient #9 during a trial of training A at the end of Stage II (i.e., after 60 training trials were performed) with the audiovisual stimulus applied at *p* = 40°. The figure shows the position of the gaze and the overall activation profile in the areas A, R, V1, E, SC at 4 different time instants (represented by lines having different colors) during the trial, together with the temporal profile of the input to SG, SG activity and gaze position [i.e., g(t)]. In order to better emphasize the shift of activity in each area of the sensory module during oculomotor response, x-axis (neuron preferred retinal position) has been restricted between −20° and +90°. The detection threshold (=0.2) and the saccade threshold (=0.65) are denoted by horizontal dashed gray lines in the visual areas and in the SG plot, respectively. Saccade onset occurs at *t* = 77 ms as a consequence of the reinforced synapses along the pathway R-SC-SG and of the simultaneous presence of the auditory stimulus. Due to the limited duration of the stimulus (100 ms), the gaze moves by only a few degrees (8° in this case) before stimulus removal.

For clarity, an example of a training trial at the end of Stage II, with the stimulus applied at position 40°, is shown in Figure 7. SC activation elicited by the stimulus at *p* = 40° (blue line) is augmented compared to the pre-training condition (Figure 5A), due to the reinforced synapses *W*^*SC, R*^. Higher SC activation, together with reinforced synapses *W*^*SG, SC*^, triggers the oculomotor response (at *t* = 77 ms, note the high value of the input to SG from the SC area). As gaze moves, an activation “wave” shifts along areas A, R, SC leftward the stimulated position *p*, and SG remains active during the oculomotor response; this promotes the strengthening of inter-areas synapses (in particular *W*^*SC, R*^ and *W*^*SG, SC*^) among neurons activated synchronously during the wave advancement. However, due to the short stimulation (100 ms), the gaze moves only by about 7°–8° before the end of the trial (i.e., before stimulus removal). Hence, in this exemplary trial, synaptic reinforcement extends leftward, from the stimulated position *p* (40°) up to ~33°, being stronger close to 40° and declining toward 33°–32°. This behavior replicates similarly when the stimulus is applied at positions 56° and 8°, the synaptic reinforcement extending leftward (with a declining trend) up to about 49° and 0°, respectively. Conversely, audiovisual stimuli at position 24° rapidly elicit visual detection and the training phase is interrupted before any oculomotor response is triggered (see section Training Paradigms and Learning Rules), not producing leftward extension of synaptic reinforcement. Importantly, since the stimulation at *p* = 24° triggers E activation too, also synapses reciprocally connecting SC and E (*W*^*SC, E*^, *W*^*E, SC*^) reinforce around that position.

**Figure 8:**
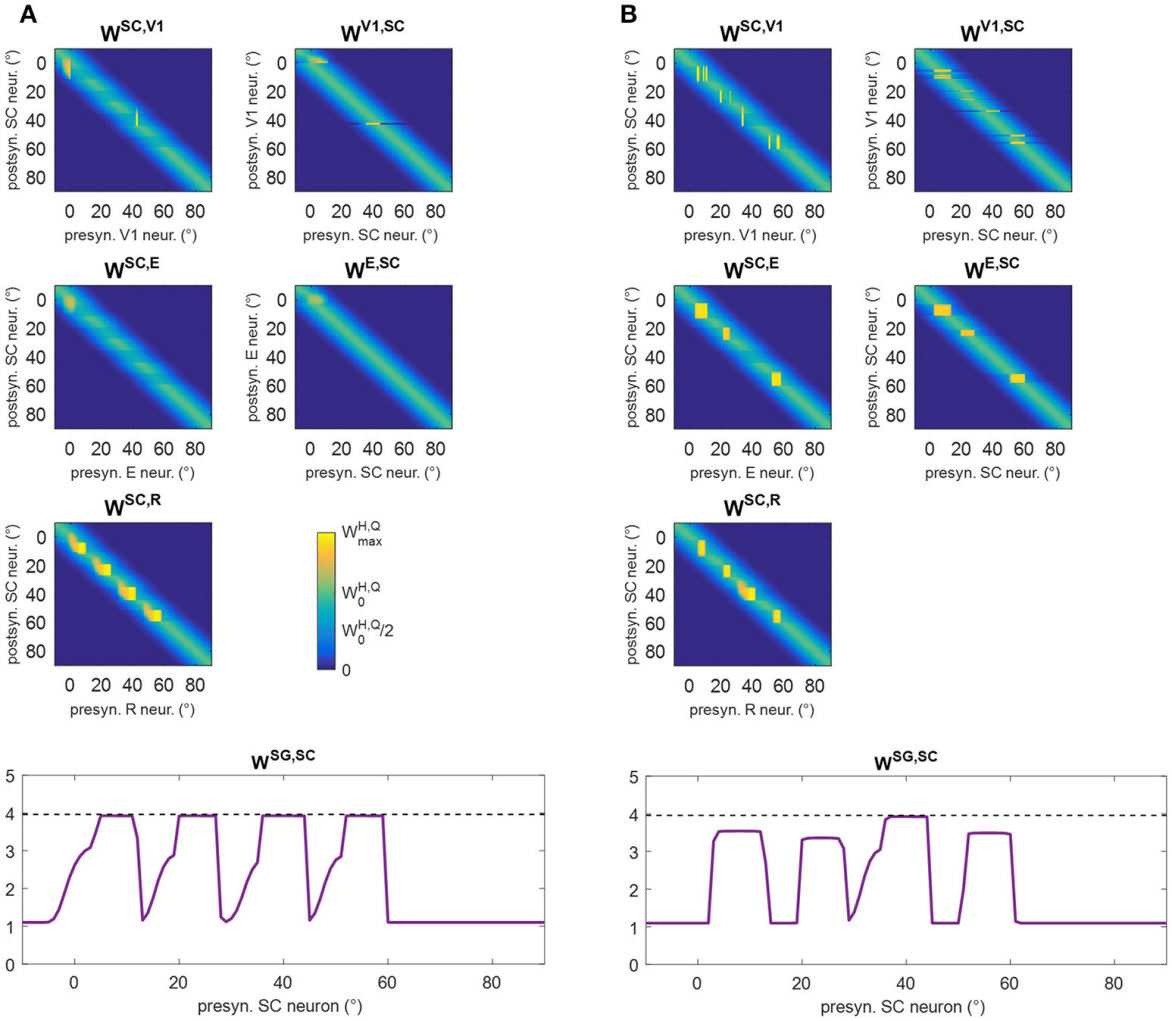
Pattern of the most significant synapses at the end of training A in patient #1 **(A)** and in patient #19 **(B)**. The meaning is the same as in Figure 6.

These behaviors explain the synaptic modifications at subsequent stages of the training (Stages III and IV) in the analyzed patient (Figure 6). Moreover, these results generalize to any simulated patients, as shown in Figure 8. The figure displays post-training synapses (in the final configuration) for other two exemplary patients (patient #1 with an isolated spared neuron, and patient #19 with several spared neurons). Patient #1 (Figure 8A) exhibits similar synaptic arrangement at the four stimulated positions, i.e., reinforcement of synapses *W*^*SC, R*^ and *W*^*SG, SC*^ that extends by about 7°–8° leftward, with a declining trend. Patient #19 (Figure 8B) exhibits this same arrangement at position 40°; at positions 8°, 24°, and 56°, where spared V1 clusters exist, reinforcement of synapses *W*^*SC, R*^ and *W*^*SG, SC*^ remains more confined, and strong reinforcement of synapses *W*^*SC, E*^ and *W*^*E, SC*^, as well *W*^*SC, V*1^ and *W*^*V*1, *SC*^, occurs too (see Figure S2 for changes in all inter-area synapses for these two patients).

According to the previous description, two circuits are mainly involved in synaptic modifications. (a) The retino-SC-motor circuit, R-SC-SG, mainly mediating a compensatory, oculomotor-related mechanism: this circuit reinforces mostly at the positions stimulated during training (8°, 24°, 40°, 56°) and, to a lower extent, at close leftward positions. (b) The retino-collicular-cortical loop, R-SC-E, mainly mediating a restitutive mechanism; this circuit reinforces at the stimulated positions in case clusters of spared V1 neurons are present. After training, visual performances may depend on the engagement of these different circuits and on their reinforcement level.

**Figure 9:**
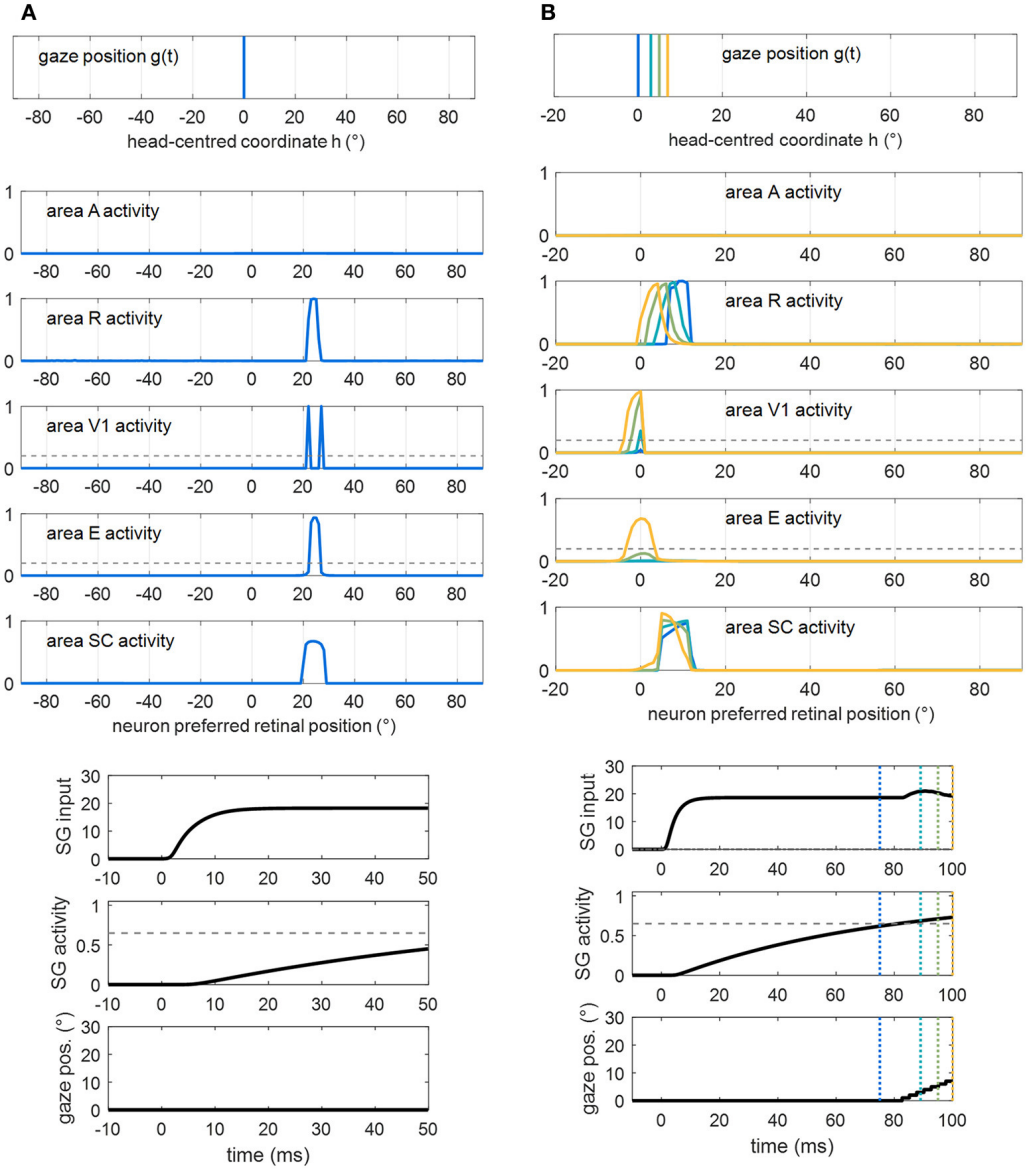
Examples of successful visual detection after training A**. (A)** The unisensory visual stimulus is applied in a position where the restitutive R-SC-E circuit was trained (*p* = 24° in patient #9). Shortly after stimulus presentation, detection occurs (both V1 and E activation overcomes the detection threshold, dashed gray line), before the oculomotor response is triggered. Compare this figure with Figure 5B (dotted purple lines). **(B)** The visual stimulus is applied in a position where the compensatory R-SC-SG circuit was trained (*p* = 8° in patient #1), close to the intact V1 side. The oculomotor response is triggered at *t* = 82 ms and the stimulus is detected at *t* = 96 ms while approaching 0°.

In particular, two principal situations may lead to successful visual detection after training A, in Eye-Movements condition (Figure 9). First, the test visual stimulus is applied at a position where the restitutive circuit R-SC-E was effectively trained. Figure 9A shows an exemplary trial (visual stimulation applied at position 24° in patient #9). Thanks to the reinforced R-SC pathway, the stimulus significantly activates area SC; the latter elicits suprathreshold E activation, via the strengthened loop SC-E. Conscious detection occurs shortly after stimulus appearance, before any potential oculomotor response is triggered. In this case, visual detection, relying on the restitutive circuit R-SC-E, occurs also in Fixed-Eyes Condition. Second, the visual stimulus is applied at a position where the compensatory circuit was effectively trained, close enough to a visual detection region (either pre-existing, e.g., the intact side, or restored). Figure 9B shows an exemplary trial (visual stimulation applied at position 8° in patient #1). Thanks to the reinforced R-SC-SG pathway, the oculomotor response is triggered at the stimulated position (at *t* = 82 ms), and the stimulus is moved into the detection region before it disappears. In this case, visual detection, relying on the compensatory circuit R-SC-SG, does not occur in Fixed-Eyes condition. In Eye-Movements condition, visual detection fails under the following circumstances. The saccade is not triggered: this occurs either because the stimulus—due to the superimposed noise—turns out to be too low to initiate a saccade even at a position of stronger synaptic reinforcement (positions stimulated during training), or because the stimulus is applied in a position were synapses were not or mildly trained (regions in between the positions stimulated during training). The saccade is triggered but the stimulus disappears before the oculomotor response has moved it into a detection region: this occurs when the stimulus is far from a visual detection region, or even when it is close to a detection region but saccade's initiation is not early enough.

**Figure 10:**
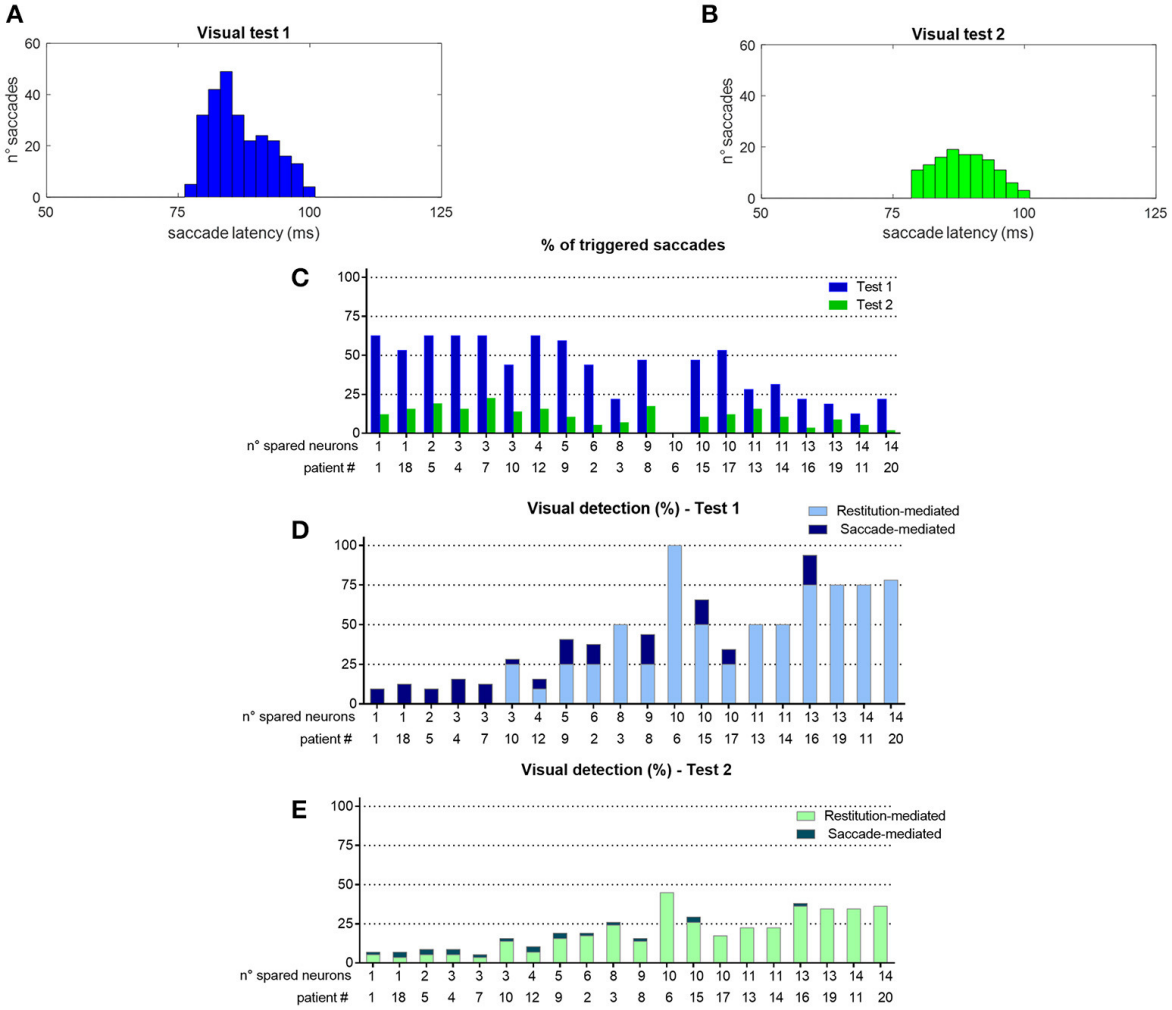
**(A,B)** Distribution of saccade latency across all patients in Visual Test 1 and Visual Test 2 (total number of saccades is 261 in Test 1 and 128 in Test 2). **(C)** Percentage of triggered saccades in each patient as a function of the number of spared V1 neurons, computed separately for Test 1 and Test 2. Computation of saccade latencies and triggered saccades does not include trials of restitution-mediated detection (where detection occurs before any potential oculomotor response). **(D,E)** Percentage of detected visual stimuli in each patient. The computation separates the detections exclusively mediated by the restitutive mechanism (restitution-mediated detections) and detections that depend on saccade execution (saccade-mediated detections, whereby the visual stimulus is moved into a visual detection region, i.e., the intact side in these simulations).

To better understand network performances after training, Figure 10 analyzes the contribution of the restitutive mechanism and compensatory mechanism to visual detection in Eye-Movements condition. Figures 10A,B shows saccade latency distribution in Test 1 and Test 2 (see section Testing Trials), across all simulated patients. Moreover, for each patient the percentage of triggered saccades (Figure 10C) and visual detections (Figures 10D,E) are reported as a function of the number of spared V1 neurons. Visual detections distinguish between those mediated by the compensatory mechanism, i.e., occurring following a saccade (saccade-mediated detections), and by the restitutive mechanism, i.e., occurring before any potential saccade (restitution-mediated detections). Computation of triggered saccades and saccade latencies does not include trials of restitution-mediated detections. After training, triggered saccades (Figures 10A,B) have latencies not lower than ~78 ms extending up to 100 ms. Total number of triggered saccades across all patients corresponds to a limited percentage of the overall stimulations (41% in Test 1 and as low as 11% in Test 2). This is evidenced in Figure 10C, showing percentage of triggered saccades in each single patient. By first looking at Test 1, we can observe that the higher the number of spared V1 neurons the lower the number of triggered saccades. Indeed, patients with larger number of spared V1 neurons more likely rely on restitution at the positions stimulated during training, making oculomotor response not necessary for detection. However, even in case of a few scattered V1 neurons (e.g., in patients # 1, 18, 5, 4, 7 where training has reinforced only the compensatory circuit), only ~60% of stimuli in Test 1 are able to trigger saccades, despite the testing stimuli are applied at the same positions stimulated during training (where training has produced maximal effect). This is the consequence of the noise superimposed over the external stimulus, occasionally reducing the stimulus effectiveness. By looking at Test 2, the percentage of triggered saccades remain lower than 25% in all patients. Indeed, while testing stimuli are applied all along the blind hemifield, only stimuli at the positions stimulated during training or slightly leftward can generate saccades, whereas at intermediate positions (in between those stimulated during training) synaptic reinforcement is lower and unable to trigger saccades (see synapses in Figure 6 or Figure 8). Figures 10D,E show that the number of saccade-mediated visual detections is drastically lower (sometimes even null) than the number of triggered saccades, in each patient and in both tests. Indeed, as most of saccades have latencies >80 ms (Figures 10A,B) and gaze velocity is 0.4°/ms, the oculomotor response can contribute to visual detection only if the stimulus triggering the saccade is at a distance not larger 8° from a visual detection region. This requirement is satisfied only by test stimuli applied at position 8° in Test 1 and at positions 4°–8° in Test 2; all other positions where saccades can be potentially triggered (24°, 40°, 56° or slightly leftward) are at least 12°–16° rightward from a possible region of visual restoration (for example, in patient #9—where visual restoration takes place at position 24°–test stimuli triggering saccades at positions 40° or 38°–36°, do not reach the restored region before their removal and remain undetected). Finally, Figures 10D,E show that restoration-mediated detections increase as the number of spared V1 neurons increases, since restitutive effects are more likely to be gained during training. Interestingly, patients with a few scattered spared V1 neurons (in particular patients # 1, 18, 5, 4, 7), that do not exhibit any visual restitution in Test 1, show a small restitutive effects in Test 2, having a few perceptions not mediated by the oculomotor response. This is the consequence of a small enlargement of the visual field at the border with the intact side (see for example synapses in patient #1, Figure 8A) so that stimuli very close to it (*p* = 4°) can be immediately detected. Of course, although Figure 10 refers to tests performed in Eye Movements condition, restoration-mediated detections equally occur in Fixed-Eyes condition.

**Figure 11:**
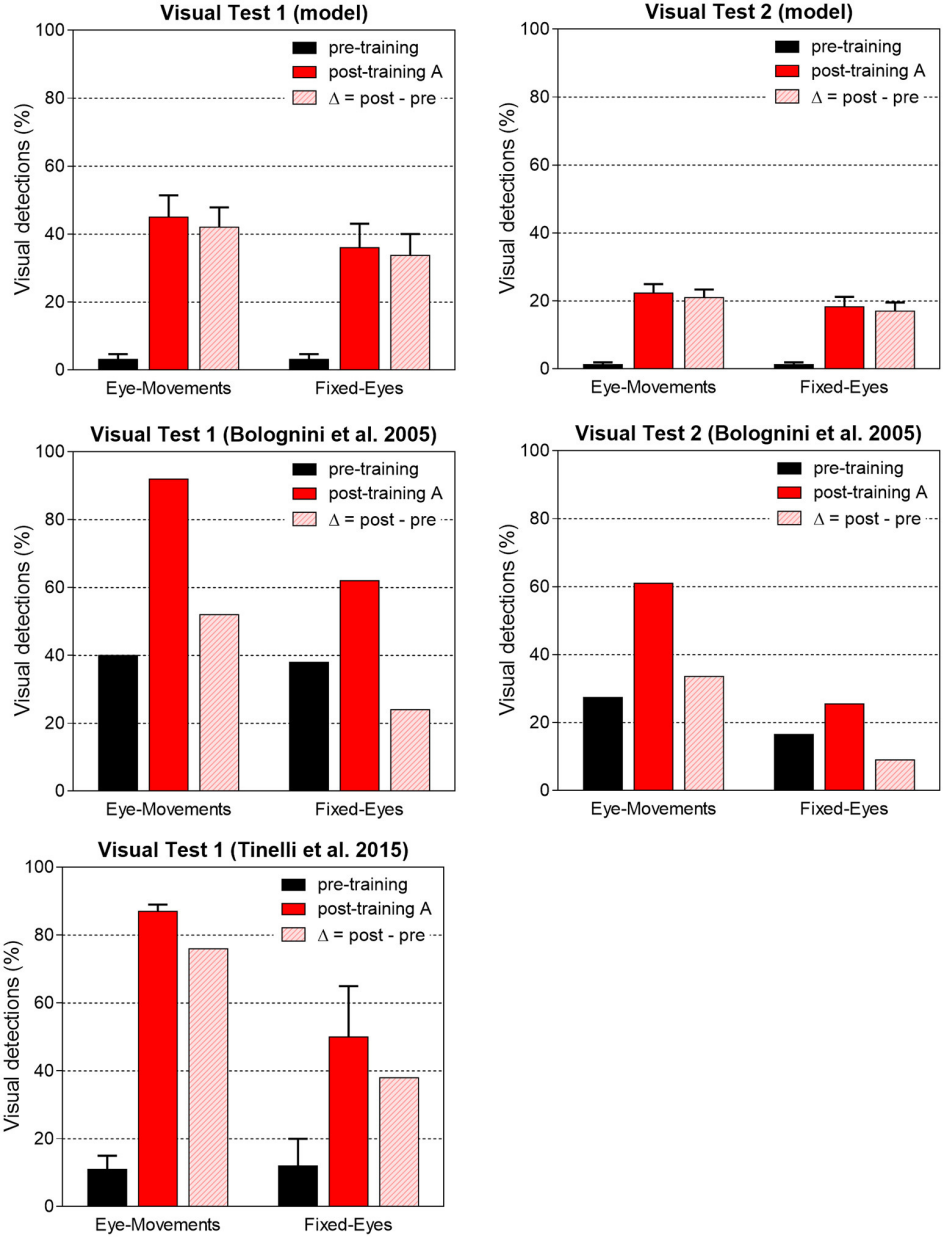
Visual detection accuracy (%, mean ± SEM) averaged on all simulated patients in Visual Test 1 and Visual Test 2 in both eye conditions before training and after training A (top histograms). The non-null detection in pre-training condition are due to the presence of a few 3-, 4-neuron spared V1 clusters around the stimulated positions that in rare instances (depending on the noise) can trigger detection. Middle and bottom histograms display average visual detection accuracy drawn from two *in vivo* studies (Bolognini et al., 2005; Tinelli et al., 2015) on patients trained with 100 ms audiovisual stimuli in Eye-Movements Condition (SEM are displayed only when available in the original papers). In each histogram, the visual detection gain acquired via the training (Δ = post–pre visual detection, dashed bar) is displayed too.

Network performances are summarized in Figure 11. The average post-training visual performances in all patients in the two visual tests are computed and presented together with *in vivo* data. Besides absolute values, the difference Δ (post-training visual detection minus pre-training visual detection) is computed to evidence the visual detection gain acquired by training and perform an even comparison between model and real data. In accord with results of Figure 10, the model predicts that amelioration is larger in Test 1 that in Test 2, in both eye conditions. Moreover, in each visual test, the model provides a larger improvement in Eye-Movements than in Fixed-Eyes condition; indeed, in Eye-Movements condition both saccade-mediated and restitution-mediated detections occur. These trends of model outcomes are in line with *in vivo* results. However, the model shows underestimation of the visual detection gain in Eye-Movements condition compared to real patients in both tests, and the advantage of Eye-Movements over Fixed-Eyes is lower than *in vivo*. This suggests that while short latency saccades, able to move the flashed (100 ms) stimulus into a visual detection region, can contribute to the observed improvement, additional mechanisms related to the oculomotor system (potentially mediated by the same circuits trained here) are likely in operation after training A (see sections Training Effects Predicted by the Model and Future Directions in the Discussion for further comments).

### Training B (audiovisual stimulation in fixed-eyes condition) and training C (visual stimulation in eye-movements condition) and post-training performances

**Figure 12:**
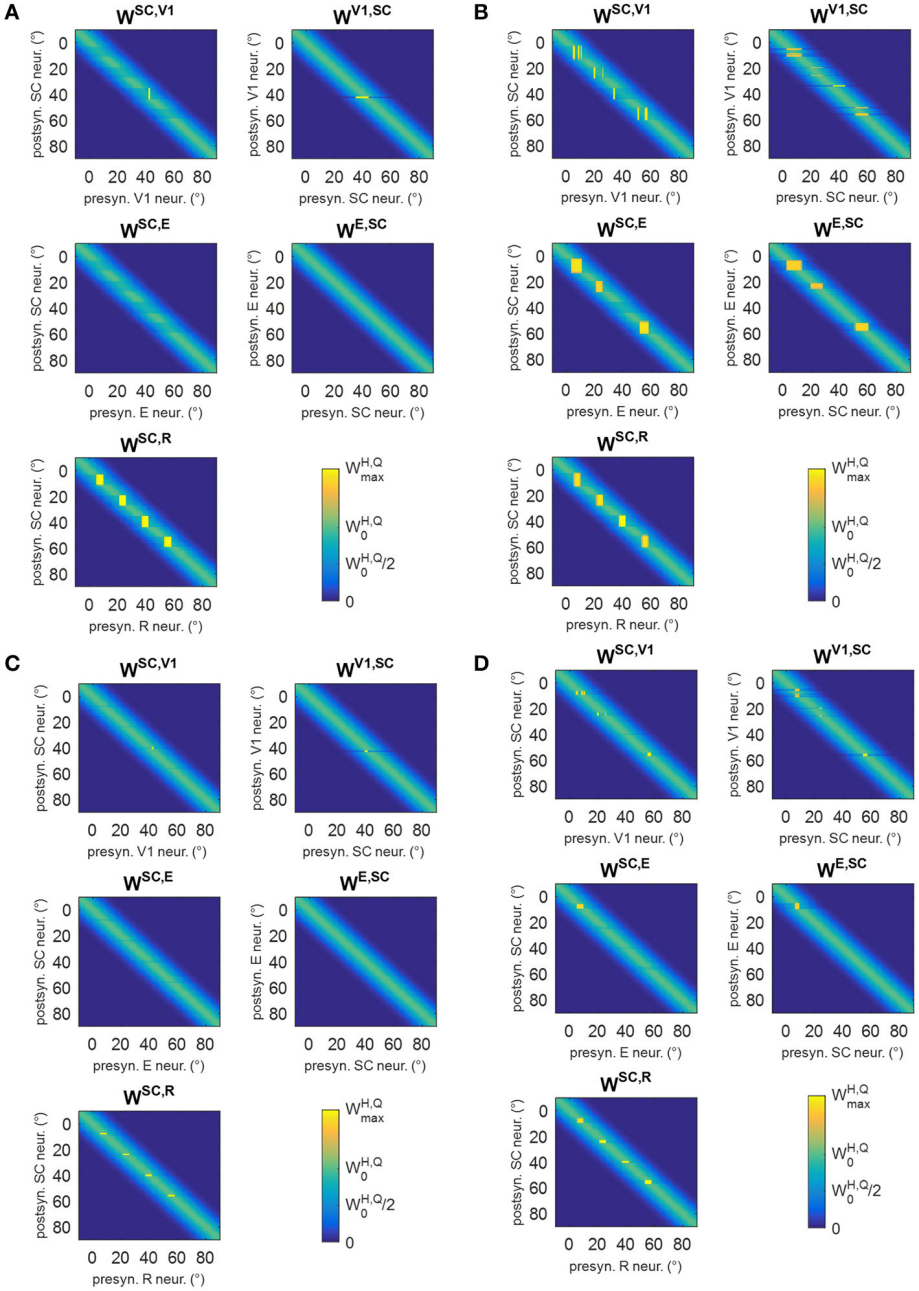
**(A,B)** Pattern of the most significant synapses at the end of training B in patient #1 **(A)** and in patient #19 **(B)**. **(C,D)** Pattern of the most significant synapses at the end of training C in patient #1 **(C)** and in patient #19 **(D)**. The meaning is the same as in Figure 6. Synapses from SC to SG are not shown since they remain at their basal value (=1.1).

In training B, audiovisual stimulations were applied while oculomotor response was inhibited, and the activations elicited by the external stimulation remained at a fixed position in the simulated neuronal areas during each training trial. Two exemplary patterns of inter-area synapses at the end of the training B are shown in Figures 12A,B (see Figure S3 for changes in all synapses). Since inputs to SG are always inhibited, synapses *W*^*SG, SC*^ do not modify. Synapses *W*^*SC, R*^ exhibit a reinforcement that remains confined around the positions stimulated during the training, without any leftward extension. As in training A, reinforcement of synapses *W*^*SC, E*^, *W*^*E, SC*^ occurs in presence of spared V1 clusters at the stimulated positions, promoting a mechanism of visual restitution at these positions.

In training C, only visual stimuli were applied and the oculomotor response was allowed. Figures 12C,D show two exemplary patterns of post-training synapses (see Figure S4 for changes in all synapses). The training promotes reinforcement of synapses *W*^*SC, R*^ in narrow regions strictly confined around the stimulated positions; indeed, because of the absence of the auditory stimulus, the visual stimulus alone produces a narrower activation in the SC. The latter does not provide a sufficient input to SG to promote reinforcement of synapses *W*^*SG, SC*^. Finally, in rare cases, where 3-neuron or 4-neuron spared clusters are present (as around position 8° in patient #19) and area E can be activated, synapses between SC and E may be reinforced.

**Figure 13:**
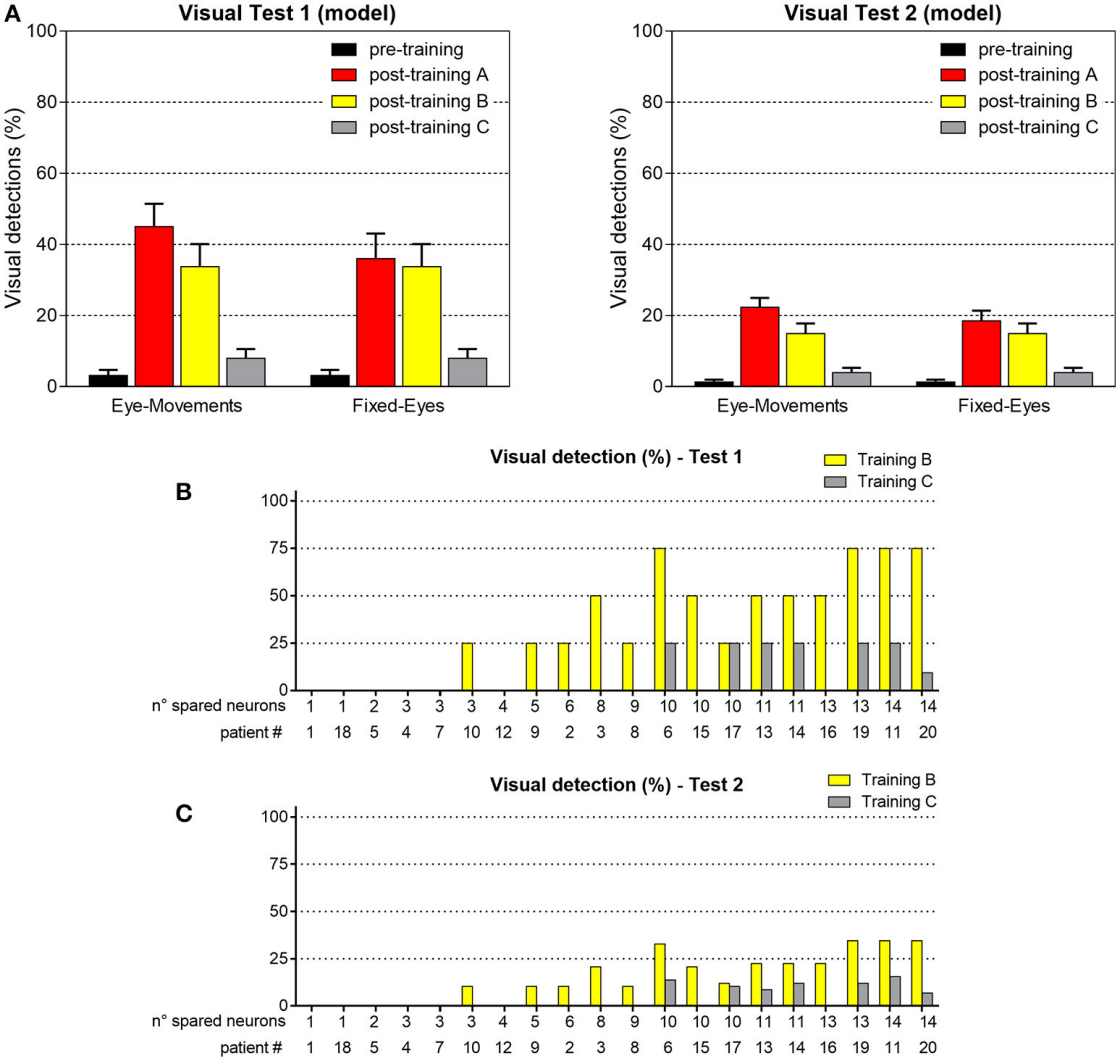
**(A)** Visual detection accuracy (%, mean ± SEM) averaged on all simulated patients in Visual Test 1 and Visual Test 2 in both eye conditions, after training B and training C (results in pre-training condition and after training A are replicated for comparison). **(B)** Visual detection accuracy (%) in each patient computed in Test 1, after training B and after training C. **(C)** Visual detection accuracy (%) in each patient computed in Test 2, after training B and after training C.

Figure 13 shows visual performances obtained in the two visual tests after training B and C. After training B, amelioration relies exclusively on the restitutive mechanism mediated by the circuit R-SC-E: visual performance remains the same regardless eye condition and almost corresponds to the value obtained in Fixed-Eyes condition after training A. After training C, the low improvement arises from the rare positions (with 3- or 4-neuron spared clusters) where the visual training has promoted visual restitution. For clarity, the % of visual detection for each patient is reported too.

## Discussion

The mechanism of multisensory integration becomes particularly beneficial when single sensory modalities are degraded or the corresponding sensory channel is damaged. The ability to integrate inputs of different modalities, especially when they are presented in temporal and spatial proximity, can enhance the unisensory processing, increasing the detectability of the weak component and its likelihood in initiating motor responses. Hemianopic patients have been shown to benefit from audiovisual stimulation not only in an online temporary way (Frassinetti et al., 2005) but also in an offline long-lasting way (Bolognini et al., 2005; Passamonti et al., 2009; Dundon et al., 2015b; Tinelli et al., 2015; Grasso et al., 2016). Here, we have modified our previous model (Magosso et al., 2016), to perform a preliminary analysis of possible functional mechanisms that may contribute to improved visual orientation and detection in these patients after audiovisual training.

### The oculomotor module

Overall, the model is based on the multisensory nature of the SC and its function as a sensory-motor interface that may exert powerful influence on overt orientation behavior (Krauzlis et al., 2013). Compared to the previous version, the network has been complemented by a module implementing saccade generation. In the last decades, countless neurocomputational models of saccade-related regions (Superior Colliculus, Frontal Eye Fields, parietal cortices, cerebellum etc.) have been proposed. Most of them provide sophisticate descriptions, accounting for the existence of different types of cells and activities in these structures and reproducing saccade kinematic properties [such as non-linear saccade-amplitude velocity, skewed velocity profile, saccade endpoint errors etc.; see (Girard and Berthoz, 2005) for a review, and (Moren et al., 2013; N'Guyen et al., 2014) as more recent examples]. Reproduction of the anatomical and neurophysiological complexity of the saccadic generation system is beyond the aim of the present study, and we provide a more conceptual description of the module, maintaining its complexity at a minimum level. Indeed, for the purpose of our analysis, we are interested only in the functional motor command generated by the SC and its effects on saccade reaction time (while saccade kinematics is just imposed). Moreover, since we currently lack data of saccade execution and saccade latencies during the investigated training and testing tasks, a parsimonious frame better conforms to the exploratory character of this study, and contributes to a more straightforward comprehension of the results.

The module includes two simple sensory-motor cascades: frontoparietal (FP) areas (modeled empirically) and the SC area project to a common brainstem motor layer (SG) modeled as a single unit that triggers a saccade when it fires above a given threshold. A fundamental point in the model, supported by neurophysiology, is that the two sensory-motor loops process the incoming sensory signal with different timing. Indeed, visual latencies in frontoparietal areas are about 100 ms on average (Boch et al., 1984; Lamme and Roelfsema, 2000), significantly larger than visual response latency in SC cells [35–80 ms (Boch et al., 1984)]. Moreover, considering that electrical stimulation of SC evokes saccades with 25–30 ms time lags (Boch et al., 1984; Sparks and Hartwich-Young, 1989), exogenously-triggered SRT as low as 70–110 ms can potentially be generated via the SC-SG pathway. The previous evidence is embedded in the model by introducing a pure delay in the FP-SG cascade [notably, a similar delay value has been recently adopted in the transmission of visual information to FEF in a computational model of saccade generation and learning (N'Guyen et al., 2014)]. The longer visual response latencies in FP justify the exclusion of this block while simulating 100 ms stimulation in hemianopic patients, at least in this preliminary study. Minimum latency from visual input to motor output via the SC is implemented in the values assigned to the time constant and saccade threshold of the motor unit, which mimic a minimum SC-mediated visuomotor delay of ≈75 ms. However, the ability of producing short latency (<110–120 ms) saccades is hampered in basal condition, because of the limited synaptic strength from SC input to SG, assigned—together with the FP input—to reproduce features of SRT distributions in agreement with behavioral data (see Figures 3, 4). Crucially, in the model, since activation in SC and its efferent input to SG depend on inter-area functional connections, assumed plastic, the SC motor command and consequently the likelihood of saccade generation and saccade reaction time may significantly change because of synaptic plasticity.

### Training effects predicted by the model

The SC is the critical node mediating the effects of training. Three pathways involving the SC are affected by the simulated trainings: the afferent projections from the retina, the retino-collicular pathway (R-SC); the feedback projections to extrastriate visual areas, the colliculo-extrastriate pathway (SC-E); the efferent projections to the motor unit, the SC-brainstem (SG) pathway (SC-SG). Different amount of modification is elicited by the different trainings along these three pathways, predicting different levels and type of rehabilitation.

After training A (audiovisual training with eye movements allowed), both the restitutive mechanism (via the reinforcement of the circuit R-SC-E) and the saccade-mediated mechanism (via the reinforcement of the circuit R-SC-SG) are operative. Accordingly, and at variance with Training B and Training C (see Figures 11, 13), training A provides higher visual performances in Eye-Movements than Fixed-Eyes Condition, in line with *in vivo* results (although the advantage of eye movements is not as large as in real studies, see also below). Indeed, the trained R-SC-SG pathway promotes production of short-latency (<100 ms) saccades toward unseen visual stimuli in the blind hemifield so they can occasionally reach a visual detection region before their removal. That the retina-SC-brainstem pathway (the shortest way from incoming visual stimulus to the oculomotor plant) can induce short latency “express” saccades and be trained for their prolific production is supported by several experimental and theoretical studies (Edelman and Keller, 1996; Isa and Kobayashi, 2004; Bibi and Edelman, 2009; N'Guyen et al., 2014; Knox and Wolohan, 2015). In humans, express saccades are commonly defined as those with latencies spanning 75–110 ms, or 80–130 ms (65–100 ms in monkeys) (Edelman and Keller, 1996; Bibi and Edelman, 2009; Knox and Wolohan, 2015), saccades with shorter latencies being considered anticipatory rather than stimulus-elicited. Recent studies (Isa and Kobayashi, 2004; Bibi and Edelman, 2009; N'Guyen et al., 2014) associate their production to an increased responsiveness of the SC-mediated saccadic system to the retinal input so that the shortcut retina-SC-brainstem becomes sufficient to trigger a saccade, without the involvement of other saccade-related cortical areas (e.g., FEF). According to the model, after training A, patients are able to perform saccades with latencies distributed in the range 75–100 ms, peaking around 85–90 ms. Similar distribution have been observed in real human subjects performing express saccades (Bibi and Edelman, 2009; Knox and Wolohan, 2015) (it is worth noticing that SRT distribution in Figures 10A,B necessarily stops at 100 ms since we limit to consider saccades triggered within 100 ms stimulus presentation). We acknowledge that model post-training SRT derive from the values of the training parameters (in particular, maximum saturation value of synapses *W*^*SG, SC*^), set by assuming a priori that, after training, patients can be able to generate saccades toward the blind hemifield with SRT <100 ms. This assumption requires comments, as actually such short latency saccades—although observed in human subjects and therefore within the limit of neurophysiological plausibility—have not been reported yet in real hemianopic patients. Indeed, experimental studies investigating reflexive saccade parameters in hemianopia (Fayel et al., 2014; Ten Brink et al., 2015), found saccade latencies of 400–500 ms in unimodal visual condition (Fayel et al., 2014) and 200 ms in audiovisual condition (Ten Brink et al., 2015). However, these studies refer to patients not specifically subjected to training procedures, hence they may correspond to our simulated patients in pre-trained conditions. Interestingly, untrained simulated patients are unable to perform reflexive saccades toward unimodal visual stimuli (Fayel and co-authors right questioned the genuineness of their visual reflexive saccades, suggesting they may actually be voluntary saccades), and perform saccades toward audiovisual stimuli with mean latency of 180 ms (omitted results) in agreement with data by Ten Brink et al. (2015). Conversely we lack saccade parameter measurements in the trained patients performing the two visual tests, for a direct comparison with our simulated post-training SRT. While this direct comparison cannot be performed, we are still aware that our model assumes a huge improvement promoted by the training in the patients (from 180 ms SRT in the best case before training to 75–100 ms after training). This hypothesized improvement is much larger than the average improvement (20–30 ms) in SRT observed in healthy subjects after they were engaged in specific visual trainings promoting production of express saccades (Bibi and Edelman, 2009; Knox and Wolohan, 2015). Our assumption of such a strong effect of training in patients is justified by the exploratory character of our study, further stressing that systematic measurements of SRT in real patients after the investigated audiovisual training are not available yet, and oculomotor mechanisms subserving the observed improvement are still largely unclear. Therefore, in our model, we hypothesized a maximum benefit of the audiovisual training on SRT (within the limit of neurophysiological plausibility)—an hypothesis that cannot be totally disregarded a priori, especially considering that multisensory integration is maximally beneficial when one sensory modality is impaired—and we tested whether and to what extent post-training capabilities of performing short-latency saccades can explain the observed improvement. By possibly predicting differences between simulated and *in vivo* performances, the model may provide cues on other oculomotor-related mechanisms (that may even rely on the same circuits as those trained here) potentially involved. Indeed, Figure 11 indicates that short latency saccades are unable by themselves to provide post-training improvements in Eye-Movements condition as high as those observed *in vivo*, not only in Test 2 but also in Test 1. That is, this mechanism not only is insufficient to explain improvement generalization to locations not directly trained, but also to reproduce the high improvement observed at the trained locations. Indeed only a limited percentage of test stimuli elicit saccades and, among the triggered saccades, only a small proportion leads to detection, because of the brief stimulus duration (Figure 10). Of course, by further increasing the maximum saturation value for synapses *W*^*SG, SC*^, the model would provide higher visual performances as the proportion of triggered saccades would increase and their latency would decrease. However, this would lead to an implausible SRT distribution, strongly unbalanced toward the lower limit of 75–78 ms. We also tested whether, simulating patients with higher pre-training visual performances (closer to those of real patients), e.g., due to islands of intact vision inside the blind hemifield, could increase post-training Eye-Movements performances. Results (see Table S1 and Figure S5) show that the new set of simulated patients still underestimates the visual detection gain in Eye-Movements condition. Accordingly, model results suggest that short-latency saccades, in case they actually occur, can contribute only partially to the observed improvement and that other oculomotor-related mechanisms are operative too (see also section Future Directions).

Audiovisual training both in Eye-Movements condition (Training A) and in Fixed-Eyes condition (Training B) elicits restorative effects. This is an important novel prediction emerging from model simulations. Restorative effects are implemented via reinforcement of the R-SC-E pathway; reinforcement of this circuit is conditional on the presence of survived clusters of V1 neurons, independently of eye condition. Audiovisual stimulation promotes restorative effects also in case of small (2-neuron) spared V1 clusters thanks to the SC multisensory enhancement, able to activate area E above threshold (see for example Figure 5B blue lines). The restorative effects predicted by the model are able to interpret the post-training amelioration in tests performed in Fixed-Eyes condition by real patients, an effect not ascribable to oculomotor strategies. Of course, in training A, restorative effects and oculomotor compensation operate together providing advantages in Eye-Movements condition compared to training B (see Figure 13).

Unisensory visual training (Training C) produces reinforcement along the R-SC pathway but limitedly to a reduced number of retinal afferents (Figures 12C,D), because of the absence of SC multisensory enhancement. At variance with audiovisual training B (promoting restitution even in case of 2-neuron spared clusters), the SC-E pathway is strengthened only in case clusters with 3–4 spared V1 neurons (regions of residual low vision) are accidentally present around the positions stimulated during the training. Indeed, because of the absence of SC multisensory enhancement, only clusters with at least 3 or 4 (but not 2) spared V1 neurons can activate E above threshold, so that the circuit SC-E can be reinforced. Regions with 3- or 4-neuron spared V1 clusters correspond to regions of residual low vision, that give rise to non-null pre-training visual performances in the simulated patients (Figure 11 upper plots). After Training C these positions regain visual sensitivity (restitutive effect); however, this occurs pretty rarely in our simulated patients (e.g., around position 8° in patient #19 or around position 56° in patient #6 etc., see Figure 13). Conversely, although the training is performed in Eye-Movements condition, the oculomotor SC-brainstem pathway remains untrained since the SC visual activity alone is unable to sufficiently activate the SG unit and promote reinforcement of synapses *W*^*SG, SC*^. Accordingly, the unimodal visual training induces only very mild rehabilitative effects, via the restorative mechanism (Figure 13). Thus in the simulated paradigms, audiovisual stimulation, producing SC multisensory enhancement, is crucial to promote more effective reinforcement in retinal afferents, more consistent E activation and possibly trigger SG activation.

It is important to specify that the visual training simulated here (Training C) is different from other visual trainings proposed in literature for rehabilitation of visual field defects, such as visual scanning training and vision restoration training (see Dundon et al., 2015a for a review). Specifically, visual scanning training (Nelles et al., 2001; Pambakian et al., 2004) stimulates compensatory oculomotor strategies by training patients to voluntarily and consciously explore arrays of visual stimuli to search for a specific target that lasts a few seconds. Such a training strongly relies on voluntary components (and on other cognitive components too, such as working memory). Conversely, here we focus only on exogenously-driven mechanisms, elicited by a flashed (100 ms long) stimulus, neglecting endogenous aspects and the effects of more salient/longer visual stimulations. The model prediction that such a visual training paradigm is unable to elicit oculomotor compensatory strategy agrees with data in real patients who did not exhibit improvement in visual oculomotor performances after a visual training similar to the one simulated here (Passamonti et al., 2009). On the other hand, vision restoration training (Sabel et al., 2004) takes advantage of areas of partial visual detection, typically at the border between intact and blind zones. Systematic visual stimulation of these areas has been shown to produce enlargement of visual field border. Interestingly, although the model is not used to mimic specifically this type of training, simulation results after Training C predicting visual restoration in regions of 3-, 4-neuron spared V1 clusters (regions of low vision), suggest that the reinforcement of the retina-SC-extrastriate pathway is a likely mechanism involved in vision restoration training.

Finally, it may be of value mentioning that the model would predict no rehabilitative effects in case of a unimodal auditory training (trivially in the model, a unisensory auditory training prevents SC neurons from regaining visual responsiveness, thus visual performances remain unaffected by the training, omitted results). Inefficacy of a unisensory auditory training is supported by a recent study (Jiang et al., 2015) showing that only audiovisual (but not auditory alone) orientation training in V1 lesioned animals boosted re-emergence of visual responsiveness in deep SC multisensory neurons that translated into recovery of orientation behavior.

### Future directions

The model hypothesizes that production of short-latency saccades is a possible oculomotor mechanism promoted by training. Comparison between model and *in vivo* results in post-training tests exhibits that the model tends to underestimate visual performances in Eye-Movements condition, suggesting that other oculomotor-related mechanisms, not included in this study, may take place. In the following, we tentatively propose another mechanism that may contribute to post-training oculomotor compensation of visual field loss, and that may be addressed in future studies.

The model assumes that once a saccade is initiated, neurons' RF updates dynamically, shifting across different locations in the external space to keep retinotopic alignment. Accordingly, when a saccade is generated toward the position of an external stimulus, a visual neuron in the model begins to respond only after the saccade brings the stimulus into its RF, and only if the stimulus is still present at that position. Actually, visual cells have been found in frontal, parietal and extrastriate cortex (Duhamel et al., 1992; Nakamura and Colby, 2002), able to respond to a stimulus that is expected to be brought into their receptive field (RF) by an eye movement, before the eye movement has been initiated and before the stimulus has arrived into their RFs. These cells respond even if the stimulus is no longer present by the time the saccade is initiated, as it occurs in case of flashed stimuli (50, 100 ms long). In other words, these cells remap their visual RFs to future locations in anticipation of an impending saccade. Pre-saccadic remapping is assumed to depend on a signal related to eye movement command (corollary discharge) distributed by saccade-related areas, in particular the SC (Sommer and Wurtz, 2008; Hall and Colby, 2011), and is considered a mechanisms implicated in visual attentional shifts (Zhao et al., 2012). A recent study (Ritchie et al., 2012) has evidenced possible implications of pre-saccadic remapping in hemianopia: patients reported increased awareness of a stimulus when they executed an instructed saccade that would bring the stimulated location into the sighted field, even though the stimulus was removed before the saccade began (however it is important to specify that only two patients were tested in that study, and further investigations are definitely required). By linking these observations to the effects of the training investigated here, we might hypothesize that the training reinforces the mechanism of pre-saccadic remapping whose benefits appear in post-training Eye-Movements condition (when motor command is not inhibited). Importantly, our model—although not directly implementing the phenomenon of pre-saccadic remapping—may provide cue on how this mechanism is reinforced or reinstantiated in patients after training. In particular the model suggests that training A, by reinforcing the R-SC-SG circuit and rendering SC neurons once again capable of transforming visual cues into oculomotor plan, may promote this further mechanism via SC corollary discharge. Indeed, the input from SC to SG (that may represents the SC motor command, i.e., the corollary discharge) elicited by a unimodal visual stimulus is lower in the untrained hemianopic patients (see Figure 5), than in healthy subjects (see Figure 3). Audiovisual training in Eye-Movements condition allows synapses from SC to SG to be reinforced; even though this reinforcement is insufficient to trigger a saccade within the time of stimulus presentation (100 ms), the input from SC to SG may reach values comparable or even higher than in healthy subjects, signaling saccade planning toward the stimulated position. Furthermore, if the mechanism of pre-saccadic remapping provides visual neurons with the ability of response facilitation not only at the future RF location but also along a continuum of positions between the current and the future RF locations [as suggested by some studies (Zirnsak et al., 2010)], this mechanism could explain both post-training improvement at positions stimulated during training (as in Test 1) and at intermediate positions (Test 2). Of course, a thorough modeling investigation of the effects of this mechanism in hemianopia before and after training is necessary.

In conclusion, the present model represents an initial step toward a quantitative formalization of the mechanisms operating during audiovisual training in hemianopia. The model predicts that the training mediates both restitutive and compensatory effects and associates them to plastic changes in specific circuits. Here, the oculomotor compensatory mechanism is assumed to be achieved only via the execution of short-latency saccades (75–100 ms), moving visual stimuli into visual detection regions. Additional mechanisms (e.g., pre-saccadic remapping), left aside in the present study but possibly mediated by the same circuits trained here, may participate in increasing the effectiveness of the oculomotor response. In future, meticulous oculomotor measurements during and after training in real patients—including both oculomotor parameters (latency, velocity, amplitude), and the relative timing between visual detection occurrence and saccade—may help to differentiate among different oculomotor-mediated mechanisms. In parallel, future model versions can be realized to predict the efficacy of other postulated oculomotor mechanisms and assess these predictions in light of existing and new experimental observations.

## Author contributions

EM: Conceived the work, developed the computational model, collected and analyzed the results, drafted, and edited the manuscript; CC and CB: Contributed to the conception of the work, analyzed the results, critically revised the work, and edited the manuscript.

### Conflict of interest statement

The authors declare that the research was conducted in the absence of any commercial or financial relationships that could be construed as a potential conflict of interest.

## Supplementary material

The Supplementary Material contains the equations describing the sensory module, Figures from S1 to S5 and Table S1.
