# Supplementary material for: Audiovisual Rehabilitation in Hemianopia: A Model-Based Theoretical Investigation
Source: Front Comput Neurosci. 2017 Dec 15;11:113. doi: 10.3389/fncom.2017.00113 (PMC5736575; doi:10.3389/fncom.2017.00113)
Supplement: Supplementary file 1 [file DataSheet1.pdf]

SUPPLEMENTARY MATERIAL

Audiovisual rehabilitation in hemianopia: A model-based theoretical investigation

Elisa Magosso, Cristiano Cuppini, Caterina Bertini

Frontiers in Computational Neuroscience

APPENDIX: Equations describing the sensory module

Each area is made up of N = 181 neurons, having preferred retinal location at a distance of 1° from each other, from -90° to + 90°, 0° representing the current gaze position (i.e. the position of the fovea). In the following, each neuron is labelled by a subscript *i* representing its preferred retinal position (*i* = -90°, -89°, …-1°, 0°, +1°, …, +89°, +90°) and by a superscript *H* denoting its area (*H* = R, V1, E, A, SC). An external stimulus applied at spatial position *p* in head-centered coordinates, at a given time *t*, will match the preferred location for neuron *i = p-g(t)* in the areas.

In order to avoid edge effects, each area in the network is considered to have a circular structure, so that the first and last neurons are virtually linked. This ensures that all neurons in each area behave in the same way regardless they are located at the border or at the center.

We use rate-coding models of neurons, which are defined via the following equations:

$$\tau \frac{dy_i^H(t)}{dt} = -y_i^H(t) + F\left(u_i^H(t)\right) \qquad Eq.S1$$

$$F\left(u_i^H(t)\right) = \frac{1}{1 + \exp(-\,(u_i^H(t) - \varphi) \cdot \xi)} \qquad Eq.S2$$

$u_i^H(t)$ denotes the input to the generic neuron i in area *H*, and $y_i^H(t)$ denotes neuron's output or neuron's activity (representing neuron's firing rate), computed from its input via a sigmoidal activation function (Eq. S2) and a first order dynamics (Eq. S1) with time constant τ. According to Eq. S2, maximum neuron's activity is normalized to 1.

The input $u_i^H(t)$ to a neuron can be generally written as the sum of three contributions:

$$u_i^H(t) = e_i^H(t) + l_i^H(t) + c_i^H(t) \qquad Eq.S3$$

The term $e_i^H(t)$ in Eq. S3, represents the external input due to the external stimulus, and it is different from 0 only in areas R and A that directly receive the stimulus. The external input in each modality is mimicked as a Gaussian function of the distance between the stimulus position (in eye-centered coordinates) and neurons' preferred position. By denoting with D the duration of the external stimulus (applied at $t = 0$) and with p its position (in head-centered coordinates), we have:

$$e_i^R(t) = \begin{cases} V_0 \exp\left(-\frac{\left(d_i^R(t)\right)^2}{2 \cdot \sigma_V^2} \right) + \eta_i^V, & 0 \le t \le D \\ 0, & otherwise \end{cases} \qquad Eq.S4$$

$$e_i^A(t) = \begin{cases} A_0 \exp\left(-\frac{\left(d_i^A(t)\right)^2}{2 \cdot \sigma_A^2} \right) + \eta_i^A, & 0 \le t \le D \\ 0, & otherwise \end{cases} \qquad Eq.S5$$

$$e_i^H(t) = 0, \quad \forall\, t, \ \ \forall\, H = V1, E, SC \qquad Eq.S6$$

$$d_i^H(t) = \begin{cases} \left|i - \left(p - g(t)\right)\right|, & if \left|i - \left(p - g(t)\right)\right| \le 90° \\ 181° - \left|i - \left(p - g(t)\right)\right|, & if \left|i - \left(p - g(t)\right)\right| > 90° \end{cases} \qquad H = R, A \qquad Eq.S7$$

$p - g(t)$ in Eq. S7 denotes the position of the external stimulation in eye-centered coordinates and definition of d_i accounts for the circular structure of each neuronal area. V_0 and A_0 are the strength of the visual and auditory stimuli. σ_V and σ_A defines the spatial reliability of the stimulus, with $\sigma_V < \sigma_A$. η_i are Gaussian noises with 0 mean and standard deviation equal to 10% of the stimulus strength.

The term $l_i^H(t)$ in Eq. S3 represents the lateral input each neuron receives from other neurons in the same area via lateral synapses. The lateral synapses are arranged according to a "Mexican Hat" disposition, obtained as the difference between an excitatory and inhibitory contribution, each mimicked as a Gaussian function of the distance between neurons' preferred positions. Hence,

$$l_i^H(t) = \sum_{k=1}^{N} L_{ik}^H \cdot y_k^H(t) \qquad \forall\, t, \qquad \forall\, H = A, R, V1, E, SC \qquad Eq.S8$$

$$L_{ik}^H = \begin{cases} L_{ex0} \exp\left(-\dfrac{(d_{ik}^H)^2}{2 \cdot (\sigma_{ex})^2}\right) - L_{in0} \exp\left(-\dfrac{(d_{ik}^H)^2}{2 \cdot (\sigma_{in})^2}\right), & d_{ik}^H \neq 0 \\ 0, & d_{ik}^H = 0 \end{cases} \qquad Eq.S9$$

$$d_{ik}^H = \begin{cases} |i-k|, & if\ |i-k| \leq 90° \\ 181° - |i-k|, & if\ |i-k| > 90° \end{cases} \qquad \forall\, i,k\ \in H \qquad Eq.S10$$

L_{ik}^H in $Eq.S9$ is the weight of the lateral synapse from the pre-synaptic neuron at position k inside area H to the post-synaptic neuron at position i inside the same area H The distance among neurons within the same area H ($Eq.S10$) is computed accounting for the circular structure of each area.

The term $c_i^H(t)$ in Eq. S3 represents the input a neuron in area H may receive from neurons in other areas via inter-area excitatory synapses. In their basal configuration (i.e. before training), they have a Gaussian shape, their strength depending on the distance between preferred retinal positions of neurons in the two connected areas. We have:

$$c_i^H(t) = \sum_Q \sum_{j=1}^{N} W_{ij}^{H,Q} \cdot y_j^Q(t), \quad \forall\, t, \qquad \forall\, H \qquad Eq.S11$$

$$W_{ij}^{H,Q} = W_0^{H,Q} \exp\left(-\frac{\left(d_{ij}^{H,Q}\right)^2}{2 \cdot (\sigma^{H,Q})^2}\right) \qquad Eq.S12$$

$$d_{ij}^{H,Q} = \begin{cases} |i-j|, & if\ |i-j| \leq 90° \\ 181° - |i-j|, & if\ |i-j| > 90° \end{cases} \qquad \forall\, i \in H,\ \ \forall\, j \in Q \qquad Eq.S13$$

$W_{ij}^{H,Q}$ in Eq. S11 is the weight of the inter-area synapse from the pre-synaptic neuron at position j in area Q to the post-synaptic neuron at position i in area H, and the first sum extends to all areas Q in the network sending projections to area H. Since area R does not receive synapses from other areas,

$c_i^R(t) = 0, \forall\, t$. $W_0^{H,Q}$ and $\sigma^{H,Q}$ in Eq S.12 are the central weight and width of the Gaussian function, and the computation of the distance $d_{ij}^{H,Q}$ among neurons' positions in different areas in eq. S13 accounts for the circular structure.

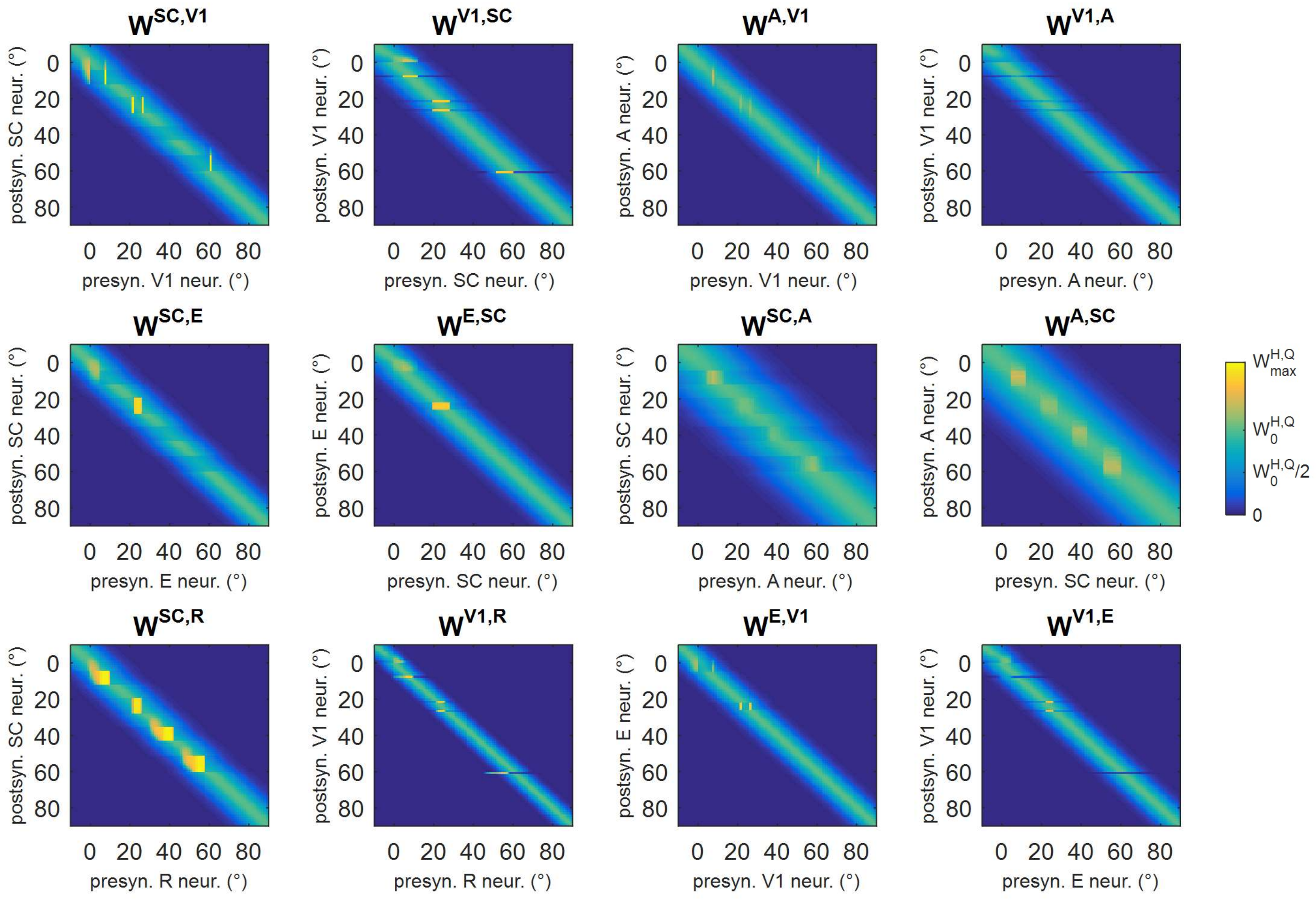

Figure S1

Figure S1: Pattern of all inter-area synapses within the sensory module at the end of training A for patient # 9 (the same as in Figure 6). In each color map, x-axis denotes the position (j) of the pre-synaptic neuron in area Q, y-axis denote the position (i) of the post-synaptic neuron in area H, and the color value at the intersection (j,i) indicates the strength of the synapse $W_{ij}^{H,Q}$. In each color map, scale color ranges between 0 and the maximum value $W_{max}^{H,Q}$ (Table 1) for the represented synapses. $W_0^{H,Q}$ is the central weight of the pre-training Gaussian pattern.

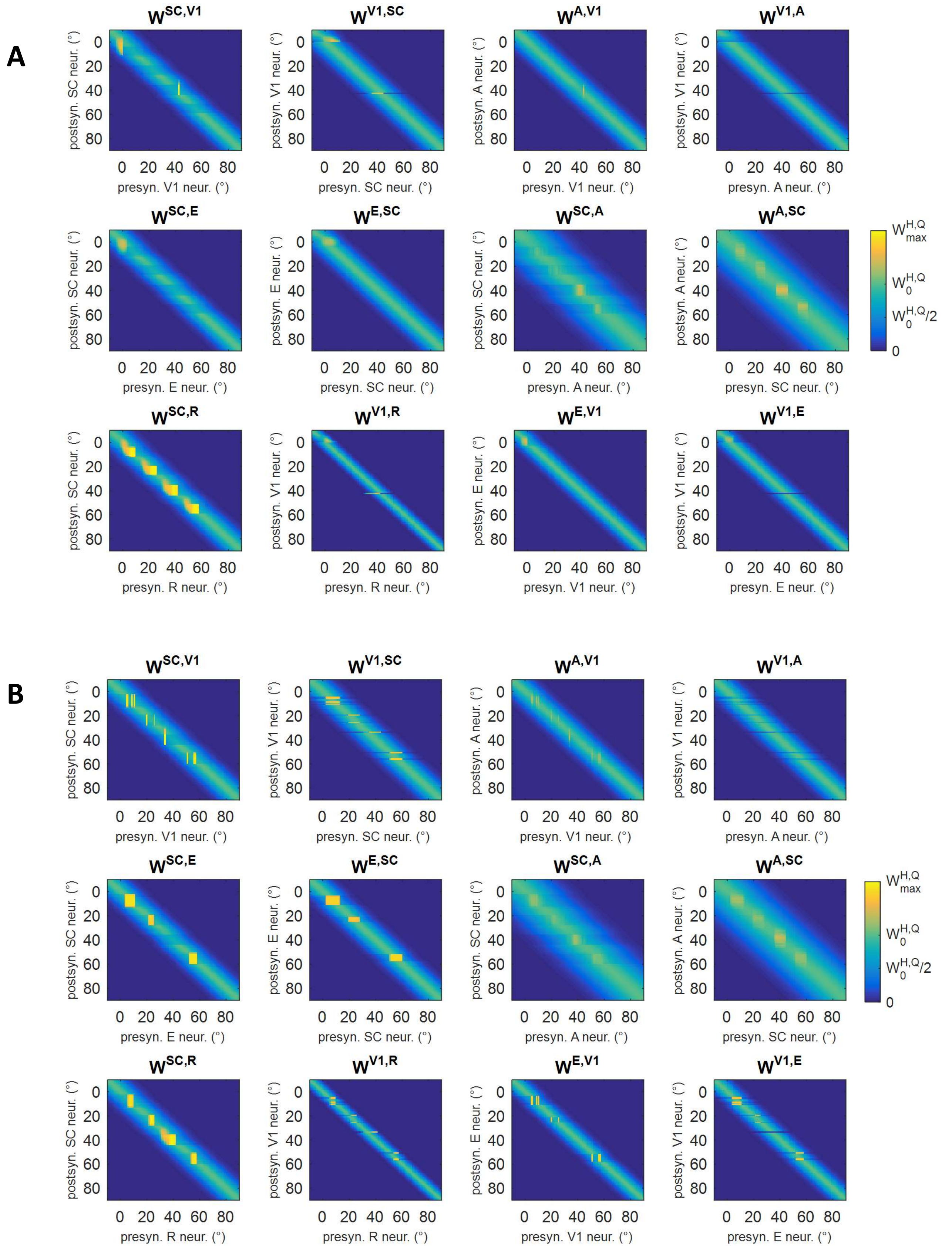

Figure S2

Figure S2 - (A) Pattern of all inter-area synapses within the sensory module at the end of training A for patient # 1 (the same as in Figure 8A). **(B)** Pattern of all inter-area synapses within the sensory module at the end of training A for patient # 19 (the same as in Figure 8B). The meaning is the same as in Figure S1.

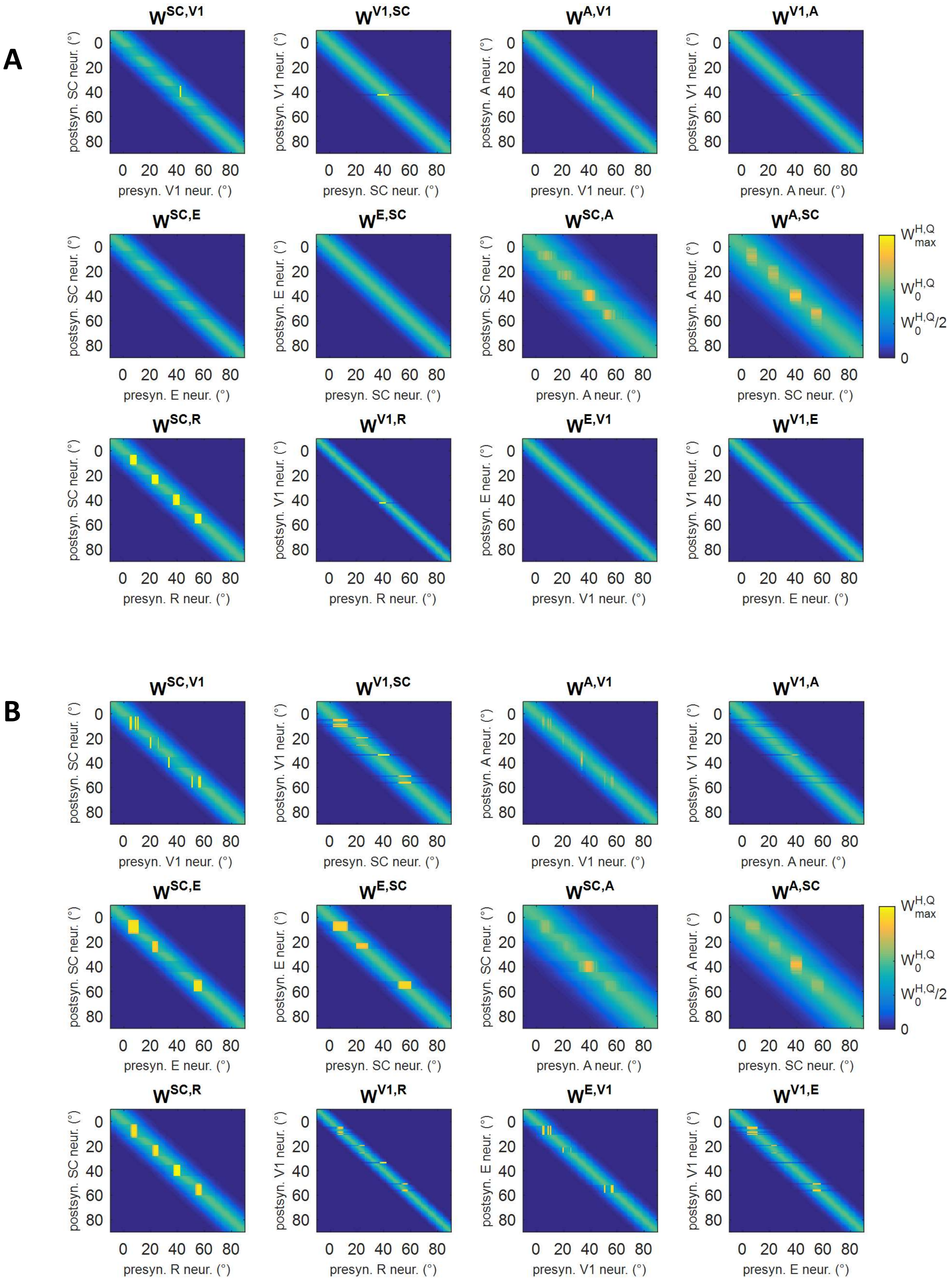

Figure S3

Figure S3 - (A) Pattern of all inter-area synapses within the sensory module at the end of training B for patient # 1 (the same as in Figure 12A). **(B)** Pattern of all inter-area synapses within the sensory module at the end of training B for patient # 19 (the same as in Figure 12B). The meaning is the same as in Figure S1.

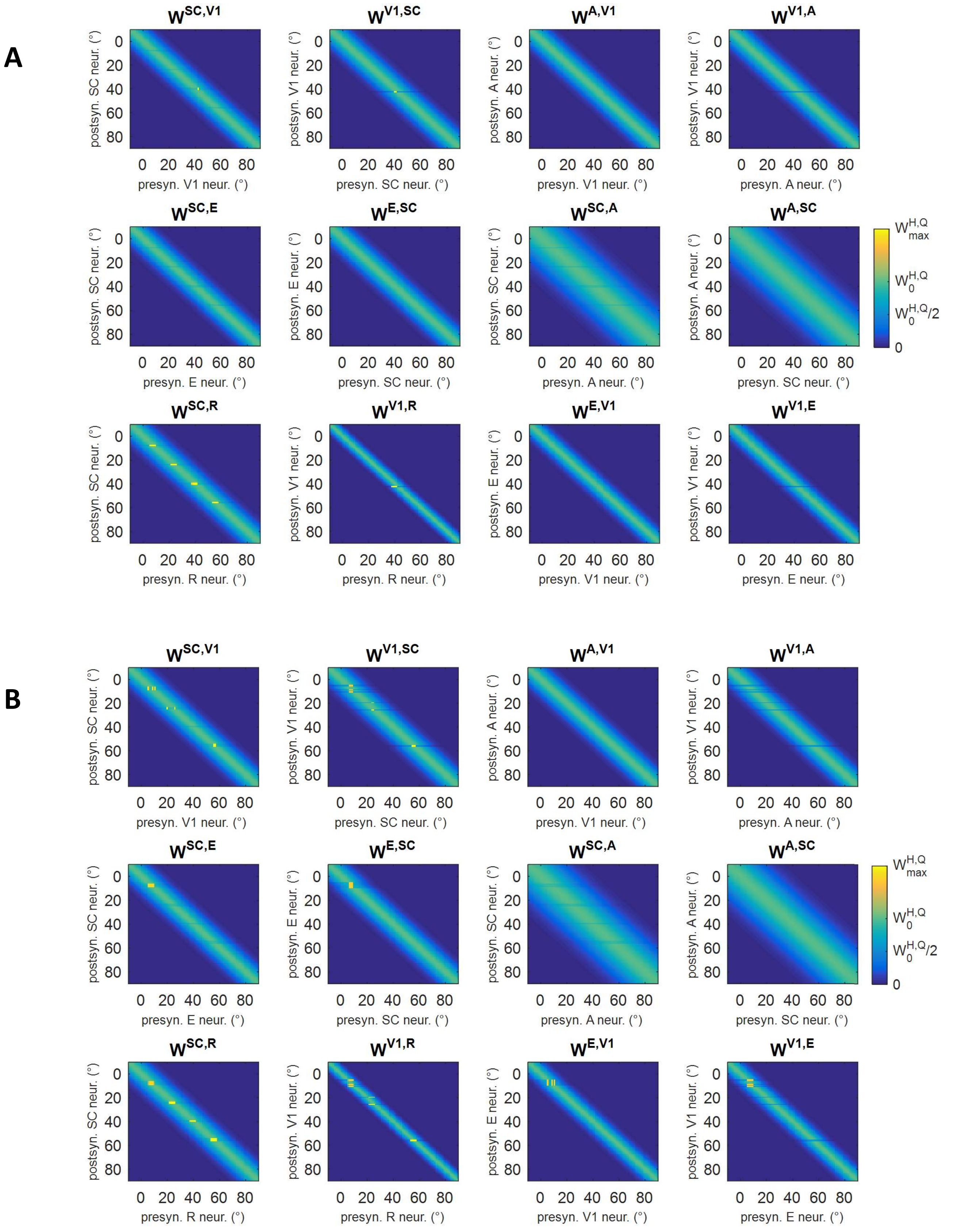

Figure S4

Figure S4 - (A) Pattern of all inter-area synapses within the sensory module at the end of training C for patient # 1 (the same as in Figure 12C). **(B)** Pattern of all inter-area synapses within the sensory module at the end of training C for patient # 19 (the same as in Figure 12D). The meaning is the same as in Figure S1.

Table S1 – Set of simulated hemianopic patients with regions of intact vision within the blind hemifield (sorted on the basis of the number of spared neurons)

Patient	Number (n) of silenced *V1* neurons in the damaged hemifield	Preferred retinal positions of the spared *V1* neurons in the damaged hemifield
1	$n = 83$	23° 24° 25° 45° 46° 47° 48°
2	$n = 78$	4° 7° 10° 11° 25° 26° 27° 28° 28° 30° 31° 32°
3	$n = 77$	10° 11° 12° 13° 14° 15° 16° 39° 41° 43° 45° 46° 47°
4	$n = 75$	1° 2° 3° 4° 5° 6° 7° 8° 9° 10° 11° 12° 13° 14° 17°
5	$n = 73$	1° 2° 3° 4° 5° 9° 11° 13° 15° 16° 42° 43° 44° 45° 46° 47° 48°

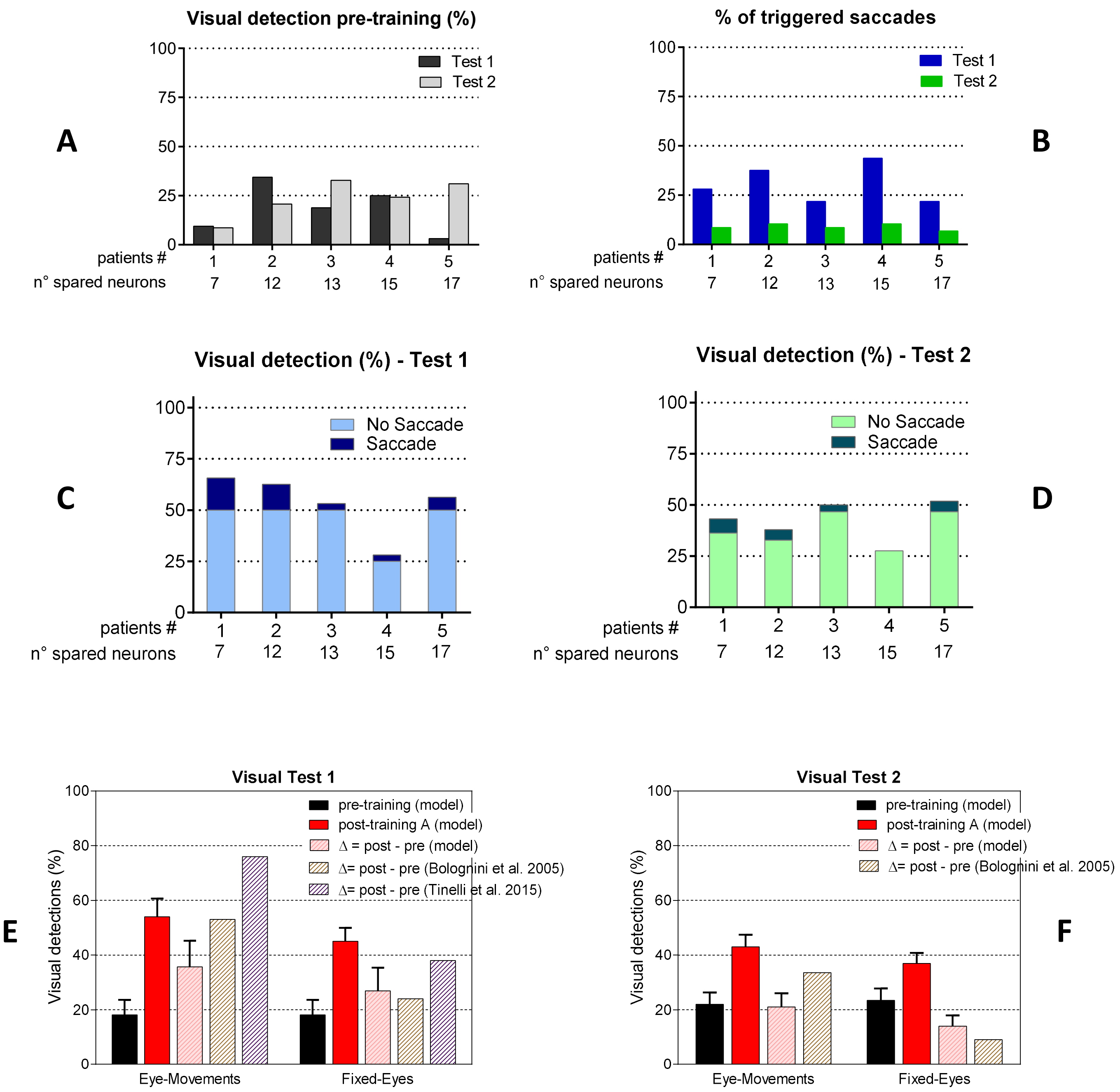

Figure S5

Figure S5 - Simulations performed on the set of 5 patients reported in Table S1. Patients are characterized by islands of low or intact vision within the blind hemifield, formed by clusters of close or contiguous spared V1 neurons, rather than having scattered spared V1 neurons as in patients reported in Table 2 (main text). Panel A refers to pre-training condition. Panels B-F display results after the patients have been subjected to training A (see main text). **(A)** Pre-training visual performances in both visual tests computed for each patient. **(B)** Percentage of triggered saccades after training, in each patient. Since patients may have islands of intact vision at the stimulated position or islands of low vision (that regain visual restitution), percentage of triggered saccades remains limited as patients rely on oculomotor mechanism only to a lower extent. In Test 2 (similarly as in the other simulated patients, see main text), percentage of triggered saccades is lower than in Test 1 because of the lower synaptic reinforcement at intermediate positions (not directly stimulated during training). **(C)** and **(D)** Percentage of visual detections in the two tests in each single patient, computed by separating detections mediated by saccade and detections that occur without the need

of oculomotor response (the latter include also detections due to islands of intact vision). Saccade-mediated detections remain a limited fraction of the triggered saccades. The reasons for the limited advantage of eye movements in post-training performances are due to the strict requirements that the visual stimulus must meet for being detected via a saccade (as in the set of patients examined in the main text): i) The visual stimulus must be applied at a position where the synapses are strongly reinforced (e.g. at or very close to the positions stimulated during training) and the noise superimposed over the external stimulus should favor high activation in the retinal area (and in SC), so that the saccade is triggered; ii) The visual stimulus triggering the saccade must be close enough (within 8° distance from a detection region); iii) The saccade must be triggered early enough so to move the stimulus into the detection region before its removal. Let's consider, for example, patient #5 in Table S1: he has islands of intact vision in the ranges 1°-5° and 42°-48° and region of low vision in the range 6°-16°. After training, he regains visual restitution at position 8° and 40°. Undetected visual stimuli at position 56° or 54° (where synapses had strongly reinforced) can trigger saccades, but only rarely they reach the intact region at 42°-48°, so they mainly remain undetected. A similar effect occurs for stimuli at position 20°-24°; they may occasionally trigger saccades (especially at position 22°, 24°) but only exceptionally elicit detection reaching the low vision region at 6°-16°. **(E)** and **(F)** Visual detection accuracy (%,) averaged (mean ± SEM) on the 5 simulated patients in Visual Test 1 and Visual Test 2 in both eye conditions, before training and after training A. The visual detection gain acquired via the training (Δ = post – pre visual detection) is displayed too and compared with visual detection gain drawn from in vivo studies (Bolognini et al., 2005; Tinelli et al. 2015). In both tests, the model underestimates the visual detection gain in Eye-Movements condition, coherently with results obtained in the other set of patients (main text), suggesting that other oculomotor mechanisms, beyond the execution of short-latency saccades, may contribute to the improvement observed in vivo.
